# Identification of positive selection in genes is greatly improved by using experimentally informed site-specific models

**DOI:** 10.1186/s13062-016-0172-z

**Published:** 2017-01-17

**Authors:** Jesse D. Bloom

**Affiliations:** Division of Basic Sciences and Computational Biology Program, Fred Hutchinson Cancer Research Center, 1100 Fairview Ave N, Seattle, 98109 WA USA

**Keywords:** Deep mutational scanning, Phylogenetics, Substitution model, Diversifying selection, dN/dS

## Abstract

**Background:**

Sites of positive selection are identified by comparing observed evolutionary patterns to those expected under a null model for evolution in the absence of such selection. For protein-coding genes, the most common null model is that nonsynonymous and synonymous mutations fix at equal rates; this unrealistic model has limited power to detect many interesting forms of selection.

**Results:**

I describe a new approach that uses a null model based on experimental measurements of a gene’s site-specific amino-acid preferences generated by deep mutational scanning in the lab. This null model makes it possible to identify both diversifying selection for repeated amino-acid change and differential selection for mutations to amino acids that are unexpected given the measurements made in the lab. I show that this approach identifies sites of adaptive substitutions in four genes (lactamase, Gal4, influenza nucleoprotein, and influenza hemagglutinin) far better than a comparable method that simply compares the rates of nonsynonymous and synonymous substitutions.

**Conclusions:**

As rapid increases in biological data enable increasingly nuanced descriptions of the constraints on individual protein sites, approaches like the one here can improve our ability to identify many interesting forms of selection in natural sequences.

**Reviewers:**

This article was reviewed by Sebastian Maurer-Stroh, Olivier Tenaillon, and Tal Pupko. All three reviewers are members of the *Biology Direct* editorial board.

**Electronic supplementary material:**

The online version of this article (doi:10.1186/s13062-016-0172-z) contains supplementary material, which is available to authorized users.

## Background

An important goal of biology is to identify genetic modifications that have led to evolutionarily significant changes in phenotype. In the case of protein-coding genes, this means identifying mutations that were fixed by selection to alter properties such as the activity of enzymes or the antigenicity of viral proteins.

This goal is challenging because not all mutations that fix do so because they confer beneficial phenotypic effects that are selected by evolution. Sometimes mutations fix because they adaptively alter phenotype, but mutations also fix due to forces such as genetic drift or hitchhiking. Therefore, it is difficult to examine gene sequences and pinpoint specific mutations that have changed evolutionarily relevant phenotypes. As Zuckerkandl and Pauling [1] noted a half-century ago:

*[Many] substitutions may lead to relatively little functional change, whereas at other times the replacement of one single amino acid residue by another may lead to a radical functional change... It is the type rather than number of amino acid substitutions that is decisive.*

Unfortunately, Zuckerkandl and Pauling [1] did not provide a prescription for determining the “type” of substitution that leads to phenotypic change, and such a prescription remains elusive decades later.

Because it is difficult to determine a priori which substitutions have altered relevant phenotypes, methods have been devised that compare homologous sequences to identify sites where mutations have been positively selected by evolution. The basic strategy is to formulate a null model for evolution, and then identify sites that have evolved in ways incompatible with this model. If the null model adequately describes evolution in the absence of selection for phenotypic change, then sites that deviate from the model are ones where mutations have been selected because they alter evolutionarily relevant phenotypes.

For protein-coding genes, the most widely used methods for identifying specific sites of positive selection are built around the null model that nonsynonymous and synonymous mutations should fix at equal rates. These methods estimate the rates of fixation of nonsynonymous (*dN*) and synonymous (*dS*) mutations at each codon site *r* [2–6]. The ratio *d* *N*/*d* *S* at *r* is taken as a measure of selection. If the ratio is clearly >1 then pressure for phenotypic change is favoring fixation of protein-altering nonsynonymous mutations, and the site is under diversifying selection. If the ratio is clearly <1 then nonsynoymous mutations are being purged to prevent phenotypic change, and the site is under purifying selection.

Although *d* *N*/*d* *S* methods are tremendously useful (the leading software implementations HyPhy and PAML have each been cited thousands of times [7, 8]), their underlying null model is clearly oversimplified. A random nonsynonymous mutation completely inactivates the typical protein ≈40% of the time [9]. So unsurprisingly, most genes have many sites with *d* *N*/*d* *S*<1. This finding is often of little biological value, since researchers frequently already know that the gene they are studying is under some type of protein-level constraint.

Perhaps more importantly, *d* *N*/*d* *S* methods also can fail to identify sites that have fixed adaptive mutations. For instance, T-cells drive fixation of immune-escape mutations in influenza – but because the relevant sites are under strong constraint, *d* *N*/*d* *S* remains <1 and the relative increase in nonsynonymous substitution rate is only apparent in comparison to homologs not subject to immune selection [10]. Therefore, even positive selection for adaptive mutations can fail to elevate *d* *N*/*d* *S*>1 at functionally constrained sites.

The limitations of simply comparing the rates of fixation of nonsynonymous and synonymous mutations have become especially glaring in light of deep mutational scanning experiments. These experiments, which subject libraries of mutant genes to selection in the lab and query the fate of each mutation by deep sequencing [11, 12], can measure the preference of each site in a protein for each amino acid [13]. A clear result is that sites vary wildly in their amino-acid preferences. Some sites are relatively unconstrained and prefer all amino acids roughly equally; for these sites, simply testing for *d* *N*/*d* *S*>1 is a reasonable approach for identifying positive selection. But most sites strongly prefer one or a few amino acids, so positive selection would not necessarily be expected to elevate *d* *N*/*d* *S*>1 for these sites.

**Fig. 1:**
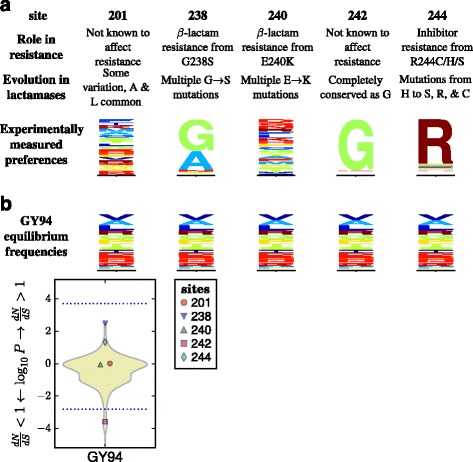
Different sites are expected to evolve differently, but *d* *N*/*d* *S* methods ignore this fact and so have limited power to detect positive selection. **a** The amino-acid preferences of five sites in TEM-1 *β*-lactamase as measured by deep mutational scanning (using the data measured with the highest concentration of ampicillin in [14]; letter heights are proportional to amino-acid preferences). Three sites experience mutations that confer extended-spectrum antibiotic or inhibitor resistance [15]. The two sites not involved in resistance are evolving in a way that seems roughly compatible with the experimentally measured amino-acid preferences, while the three sites implicated in resistance are evolving in ways that clearly deviate from the preferences (for instance, site 238 mutates from highly preferred glycine to the very low preference amino-acid serine). **b** A standard *d* *N*/*d* *S* model (the M0 variant [4] of the Goldman-Yang model [23], abbreviated GY94) assumes all sites evolve under uniform constraints. When this model is used to fit a site-specific *d* *N*/*d* *S*, no sites are deemed under diversifying selection (*d* *N*/*d* *S*>1) at a FDR of 0.05 for testing all sites, although the non-resistance site 242 is deemed under purifying selection (*d* *N*/*d* *S*<1). The violin plot shows the distribution of *P*-values for sites having *d* *N*/*d* *S*> or <1. All sites below the bottom dotted blue line are deemed to have *d* *N*/*d* *S*<1 at an FDR of 0.05. No sites have *d* *N*/*d* *S*>1 at this FDR, so the top dotted blue line indicate the *P*-value that would be needed for a site to have *d* *N*/*d* *S*>1 at a significance level of 0.05 using a Bonferroni correction. A full analysis of all sites and further details are later in the paper. See Additional file 16 for subtleties about amino-acid preferences versus equilibrium frequencies

As an example, Fig. 1 shows the amino-acid preferences of five sites in TEM-1 *β*-lactamase as measured by the deep mutational scanning of Stiffler et al [14]. Mutations at three of these sites confer antibiotic or inhibitor resistance in *β*-lactamases [15]. Inspection of Fig. 1 shows that the two sites not implicated in resistance have evolved in ways that seem roughly compatible with their amino-acid preferences measured in the lab: site 201 tolerates many amino acids in the lab and is moderately variable in nature, while site 242 strongly prefers glycine in the lab and is conserved at that identity in nature. But the three sites involved in resistance have evolved in ways that seem to deviate from their amino-acid preferences measured in the lab: site 238 substitutes from the lab-preferred glycine to the less preferred serine, site 240 repeatedly substitutes to lysine despite not strongly preferring this amino acid in the lab, and site 244 substitutes from the lab-preferred arginine to several less preferred amino acids. So given the experimentally measured preferences, it is fairly apparent that the sites where mutations contribute to antibiotic resistance are evolving in ways that deviate from the preferences measured in the lab. But as Fig. 1 shows, a *d* *N*/*d* *S* method fails to find any site with *d* *N*/*d* *S*>1 at a false-discovery rate (FDR) of 0.05. As this example shows, a null model that fails to account for site-specific amino-acid preferences can overlook sites that fix adaptive mutations.

Here I describe how the limitations of *d* *N*/*d* *S* methods illustrated in Fig. 1 can be overcome by defining selection relative to a null model established by experimentally measured site-specific amino-acid preferences. This more nuanced null model can be used to identify sites of *diversifying selection* for unusually rapid amino-acid change via a statistically principled extension to standard *d* *N*/*d* *S* methods. The more nuanced null model can also be used to heuristically identify sites of *differential selection* for unexpected amino acids. Both of these strategies ultimately seek to identify sites that are evolving differently in nature than expected from constraints measured in the lab. Although the lab measurements are undoubtedly imperfect proxies for actual selective constraints in nature, they provide a better model for evolution in nature than phylogenetic substitution models commonly used to identify positive selection in nature. I demonstrate that this is the case by analyzing four genes, and showing that the experimentally informed methods greatly outperform a standard *d* *N*/*d* *S* method at identifying sites of antibiotic-resistance and immune-escape mutations. As deep mutational scanning data become more widespread, approaches like the one here can enhance our ability to identify sites of biologically interesting selection.

## Results

### An evolutionary null model informed by experimentally measured amino-acid preferences

To remedy the limitations of *d* *N*/*d* *S* methods illustrated in Fig. 1, we formulate a description of how sites should evolve if selection in nature matches the constraints measured by deep mutational scanning in the lab. This description consists of a set of site-specific experimentally informed codon models (ExpCM). The ExpCM used here are similar but not identical to those in [16, 17]. Specifically, they differ from the model in [17] by inclusion of an *ω* parameter representing the relative rate of nonsynonymous to synonymous substitutions, and by handling the nucleotide mutation terms via an HKY85-style [18] formalism rather than the formalism in [17].

Deep mutational scanning experiments provide direct measurements of the preference *π*_*r*,*a*_ of each site *r* for each amino acid *a* (for details of how these preferences can be obtained from the experimental data, see [13]). These preferences are normalized so $\sum _{a} \pi _{r,a} = 1$. We use the preferences to define an ExpCM for each site. As is typical for phylogenetic substitution models, each ExpCM is a reversible stochastic matrix giving the rates of substitution between codons. The rate *P*_*r*,*xy*_ from codon *x* to *y* at site *r* is written in mutation-selection form as

1 $$  P_{r,xy} = Q_{xy} \times F_{r,xy}  $$

where *Q*_*xy*_ represents the rate of mutation from *x* to *y* and *F*_*r*,*xy*_ represents the selection on this mutation. The mutation terms are identical across sites, but the selection terms are site-specific.

The mutation terms *Q*_*xy*_ are given by a HKY85 model [18], and consist of a transition-transversion ratio *κ* and four nucleotide parameters *ϕ*_*A*_, *ϕ*_*C*_, *ϕ*_*G*_, and *ϕ*_*T*_ that sum to one. These *ϕ* parameters give the expected nucleotide composition in the absence of selection on amino acids; the actual nucleotide frequencies are also influenced by the selection (for this reason, the *ϕ* terms cannot simply be equated with the empirical alignment frequencies). The mutation term is:

2 $$  \begin{aligned} Q_{xy} = \left\{\begin{array}{ll} 0 & \text{\textit{x} and \textit{y} differ by \(> 1\) nucleotide,} \\ \phi_{w} & \text{\textit{x} can be converted to \textit{y} by transversion to \textit{w},} \\ \kappa \times \phi_{w} & \text{\textit{x} can be converted to \textit{y} by transition to \textit{w}.} \\ \end{array}\right. \end{aligned}  $$

The site-specific amino-acid preferences *π*_*r*,*a*_ enter the model via the selection terms *F*_*r*,*xy*_. Let A(*x*) denote the amino acid encoded by codon *x*, let *β* be the stringency parameter described in [17], and let *ω* be a gene-wide relative rate of fixation of nonsynonymous to synonymous mutations after accounting for the amino-acid preferences. Then:

3 $$  \begin{aligned} F_{r,xy} =\left\{ \begin{array}{ll} 1 & \text{if}\, \mathcal{A}(x) = \mathcal{A}(y) \\ \omega & \text{if}\, \mathcal{A}(x) \ne \mathcal{A}(y)\, \text{and}\, \pi_{r,\mathcal{A}(x)} = \pi_{r,\mathcal{A}(y)} \\ \omega \times \frac{\ln\left[\frac{\left(\pi_{r,\mathcal{A}(y)}\right)^{\beta}}{\left(\pi_{r,\mathcal{A}(x)}\right)^{\beta}}\right]}{1 - \frac{\left(\pi_{r,\mathcal{A}(x)}\right)^{\beta}}{\left(\pi_{r,\mathcal{A}(y)}\right)^{\beta}}} & \text{otherwise.} \end{array}\right. \end{aligned}  $$

The functional form relating *F*_*r*,*xy*_ to *π*_*r*,*a*_ for nonsynonymous mutations is that derived by Halpern and Bruno [19] under certain (probably unrealistic) assumptions about the evolutionary process and the relationship between the preferences and amino-acid fitnesses (see also [20–22]). Relative to the equation of Halpern and Bruno [19], Eq. 3 removes terms related to mutation (these are captured by *Q*_*xy*_) and corrects a typographical error in the denominator. The stringency parameter *β* is >1 if natural selection favors high-preference amino acids with greater stringency than the experiments used to measure *π*_*r*,*a*_, and is <1 if it favors them with less stringency. Under the assumptions of Halpern and Bruno [19], *β* is related to effective population size. Note that if *β*=0, then the substitution model defined by Eq. 1 reduces to a F1X4 version of the M0 variant [4] of the Goldman-Yang [23] model. The *ω* parameter indicates if there is a retardation (*ω*<1) or acceleration (*ω*>1) in the rate of fixation of nonsynonymous mutations relative to synonymous mutations after accounting for the preferences. In [17], it is shown that a model of the form defined by *P*_*r*,*xy*_ is reversible and has stationary state

4 $$  p_{r,x} = \frac{\left(\pi_{r,\operatorname{A}(x)}\right)^{\beta} \times \phi_{x_{1}} \times \phi_{x_{2}} \times \phi_{x_{3}}}{\sum_{y} \left(\pi_{r,\operatorname{A}(y)}\right)^{\beta} \times \phi_{y_{1}} \times \phi_{y_{2}} \times \phi_{y_{3}}}  $$

where *x*_1_, *x*_2_, and *x*_3_ are the nucleotides at positions 1, 2, and 3 of codon *x*.

The ExpCM can be used to calculate the likelihood of a phylogenetic tree and an alignment of genes using the algorithm of Felsenstein [24], which implicitly assumes that sites evolve independently. The set of ExpCM for a given gene have six free parameters: *ω*, *β*, *κ*, and three of the *ϕ*’s. The *π*_*r*,*a*_ values are not free parameters, since they are specified a priori from experimental data. The values of the six free parameters are fit by maximum likelihood.

Overall, the ExpCM describe how sites evolve if selection in nature is concordant with the amino-acid preferences measured in the lab.

### Identifying sites of diversifying selection

Having established a null model for how a gene should evolve if selection adheres to the constraints measured in the lab, we next want to identify sites that deviate from this model. Such sites are likely targets of additional selection. One such form of selection is *diversifying selection* for amino-acid change, as occurs at viral epitopes under continual pressure to escape newly generated immunity.

To detect diversifying selection, we use an approach analogous the fixed effects likelihood (FEL) method [5, 25, 26]. After fixing the tree and model parameters to their maximum likelihood values for the entire sequence, for each site *r* we fit a synonymous rate *μ*_*r*_ and a parameter *ω*_*r*_ corresponding to the nonsynonymous rate relative to the synonymous rate by replacing Eq. 3 with

5 $$  \begin{aligned} F_{r,xy} =\left\{ \begin{array}{lll} \mu_{r} & \text{if}\, \mathcal{A}(x) = \mathcal{A}(y) \\ \mu_{r} \times \omega_{r} & \text{if}\, \mathcal{A}(x) \ne \mathcal{A}(y) \text{and}\, \pi_{r,\mathcal{A}(x)} = \pi_{r,\mathcal{A}(y)} \\ \mu_{r} \times \omega_{r} \times \frac{\ln\left[\frac{\left(\pi_{r,\mathcal{A}(y)}\right)^{\beta}}{\left(\pi_{r,\mathcal{A}(x)}\right)^{\beta}}\right]}{1 - \frac{\left(\pi_{r,\mathcal{A}(x)}\right)^{\beta}}{\left(\pi_{r,\mathcal{A}(y)}\right)^{\beta}}} & \text{otherwise.} \end{array}\right. \end{aligned}  $$

and optimizing with respect *μ*_*r*_ and *ω*_*r*_. The reason that we fit *μ*_*r*_ as well as *ω*_*r*_ is to accommodate synonymous rate variation among sites; this can be important for the reasons described in [27]. The null hypothesis is that *ω*_*r*_=1. Following [5], we compute a P-value for rejecting this null hypothesis by using a ${\chi _{1}^{2}}$ test to compare the likelihood when fitting both *μ*_*r*_ and *ω*_*r*_ to that when fitting only *μ*_*r*_ and fixing *ω*_*r*_=1. The key statistic is not *ω*_*r*_ itself, but rather the difference in log likelihood (the likelihood ratio) from which we compute the P-value for rejecting the null hypothesis of *ω*=1 in favor of *ω*_*r*_>1 or *ω*_*r*_<1. The former case implies diversifying selection, while the latter case indicates a selective constraint on amino-acid change that is not adequately captured by the preferences. To account for the fact that a different test is performed for each site, we control the FDR using the Benjamini-Hochberg procedure [28]. As demonstrated below, this approach has excellent power to pinpoint sites like 238 and 244 in Fig. 1, which fix multiple nonsynonymous mutations despite being under strong functional constraint.

### Identifying sites of differential selection

Some interesting forms of selection do not cause sites to change repeatedly, but rather lead them to substitute to amino acids that are unexpected given the amino-acid preferences measured in the lab. Such sites are under *differential selection* to fix mutations different from those expected if selection in nature parallels that in the lab.

To detect differential selection, we compare the preferences measured in the lab to those that optimally describe evolution in nature. We again begin by fixing the tree and model parameters to their maximum likelihood values determined over the whole gene. We then examine the effect of allowing the preferences at each site to differ from the values measured in the lab. Specifically, denote the preferences that optimally describe evolution in nature as $\hat {\pi }_{r,a}$, with $\sum _{a} \hat {\pi }_{r,a} = 1$. Denote the differential preference *Δ* *π*_*r*,*a*_ for amino-acid *a* at site *r* as the difference between $\hat {\pi }_{r,a}$ and the experimentally measured preferences rescaled by the stringency parameter: $\Delta \pi _{r,a} = \hat {\pi }_{r,a} -\frac {\left (\pi _{r,a}\right)^{\beta }}{\sum _{a'} \left (\pi _{r,a'}\right)^{\beta }}$. If we redefine Eq. 3 by replacing (*π*_*r*,*a*_)^*β*^ with $\hat {\pi }_{r,a}$ as in

6 $$  F_{r,xy} \,=\,\left\{\!\! \begin{array}{ll} 1 & \text{if}\, \mathcal{A}(x) =\! \mathcal{A}(y) \\ \omega & \text{if}\, \mathcal{A}(x) \ne\! \mathcal{A}(y) \text{and}\, \hat{\pi}_{r,\mathcal{A}(x)} \,=\, \hat{\pi}_{r,\mathcal{A}(y)} \\ \omega \times\! \frac{\ln\left[\frac{\hat{\pi}_{r,\mathcal{A}(y)}}{\hat{\pi}_{r,\mathcal{A}(x)}}\right]}{1 - \frac{\hat{\pi}_{r,\mathcal{A}(x)}}{\hat{\pi}_{r,\mathcal{A}(y)}}} & \text{otherwise,} \end{array}\right.  $$

then we can determine the preferences that optimally describe natural evolution by optimizing with respect to $\hat {\pi }_{r,a}$ after fixing the tree and model parameters to their maximum likelihood values for the entire sequence. However, unconstrained optimization of Eq. 6 will overfit the data [29, 30]. We therefore instead optimize the product of Eq. 6 and an Eq. that regularizes the *Δ* *π*_*r*,*a*_ values by biasing them towards zero:

7 $$  \Pr\left(\left\{\hat{\pi}_{r,a}\right\} \mid \left\{\pi_{r,a}\right\}, \beta\right) = \prod_{a} \left(\frac{1}{1 + C_{1} \times \left(\Delta\pi_{r,a}\right)^{2}}\right)^{C_{2}}  $$

where *C*_1_ and *C*_2_ determine how strongly $\hat {\pi }_{r,a}$ is biased towards the experimentally measured preferences. Here I use *C*_1_=150 and *C*_2_=0.5; Eq. 7 is illustrated in Additional file 1. Effectively, this equation biases the estimated values towards the prior expectation from the deep mutational scanning, although the equation is not a true prior as we are using a maximum-likelihood rather than a Bayesian approach. Note that while the underlying rationale for regularizing the *Δ* *π*_*r*,*a*_ values is clear, the regularization implemented by Eq. 7 was chosen heuristically with the rationale that the marginal cost of shifting *Δ* *π*_*r*,*a*_ away from zero should initially be steep but then flatten somewhat, corresponding to the intuition that most sites have little differential selection, but some have a lot. However, a more statistically principled method for assessing the support for non-zero *Δ* *π*_*r*,*a*_ values is an important area for future work.

A differential preference of *Δ* *π*_*r*,*a*_>0 implies that natural evolution favors amino-acid *a* at site *r* more than expected, whereas *Δ* *π*_*r*,*a*_<0 implies that evolution disfavors this amino acid. The total differential selection at *r* is quantified as half the absolute sum of the differential preferences, $\frac {1}{2} \sum _{a} \left |\Delta \pi _{r,a}\right |$; this quantity ranges from zero to one. As demonstrated below, this approach has excellent power to pinpoint sites like 238 and 240 in Fig. 1, which fix mutations to unexpected amino acids. However, I emphasize that this test for differential selection is heuristic, and does not incorporate formal statistical significance testing.

### Choice of four genes to test approaches to identify sites of selection

To test the approaches for detecting selection described above, I selected four genes: the DNA-binding domain of yeast Gal4, *β*-lactamase, the nucleoprotein (NP) of human influenza, and the hemagglutinin (HA) of human seasonal H1N1 influenza. Previous deep mutational scanning studies have measured the effects of all mutations to these genes [14, 31–33], enabling calculation of their site-specific amino-acid preferences. For *β*-lactamase there are actually two deep mutational scanning datasets: one from Firnberg et al [34] and a more recent one from Stiffler et al [14]. As will be shown below, a likelihood-based model comparison shows that the latter of these two datasets provides a better description of *β*-lactamase evolution in nature, and so for that reason this is the *β*-lactamase deep mutational scanning dataset used in the current study. For each gene, I assembled an alignment of homologs for evolutionary analysis (Table 1).

**Table 1:** The four genes analyzed in this study

Gene	# of residues	Deep mutational scanning	Alignment details
Yeast Gal4 DNA binding domain	64	[31]	17 sequences with 87% and 59% avg and min pairwise protein identity
*β*-lactamase	263	[14]	85 sequences with 82% and 63% avg and min pairwise protein identity
Influenza nucleoprotein (NP)	498	[32]	180 sequences with 95% and 90% avg and min pairwise protein identity
Influenza H1 hemagglutinin (HA)	564	[33]	111 sequences with 95% and 87% avg and min pairwise protein identity

A great deal is known about the pressures that have shaped the evolution of all four genes. Gal4 performs a function that is conserved among homologs from widely diverged species, and does not appear to be changing phenotypically [35, 36]. However, the other three genes are undergoing adaptive evolution: *β*-lactamases evolve resistance to new antibiotics and inhibitors [15, 37], while NP and HA evolve to escape the immune response in humans [10, 38–40]. These genes therefore provide an excellent test case. Gal4 is a “negative control”: no sites in this gene should be identified as under selection to fix adaptive mutations. But an effective approach for identifying positive selection should pinpoint the sites of drug-resistance and immune-escape mutations in the other three genes.

### Experimentally informed site-specific models are vastly better descriptors of evolution

Our basic assumption is that site-specific ExpCM are a better null model for evolution than the non-site-specific models used by *d* *N*/*d* *S* methods. Prior work has shown that experimentally informed site-specific models similar to the ExpCM defined here greatly outperform non-site-specific models [16, 17, 32, 33]. To confirm this result for the ExpCM and genes here, I compared the ExpCM to the several variants [4] of the Goldman-Yang style models [23] (denoted as GY94) commonly used by *d* *N*/*d* *S* methods. I used F3X4 equilibrium frequencies for GY94, with the nine F3X4 parameters estimated by maximum likelihood. These equilibrium frequencies are *not* site-specific; this is the major difference between GY94 and ExpCM (Fig. 1).

To compare the models and perform the other analyses in this paper, I developed the software package phydms (**phy**logenetics informed by **d**eep **m**utational **s**canning; https://github.com/jbloomlab/phydms). This software interfaces with and extends Bio++ [41, 42] to enable analyses with both ExpCM and GY94 models. The analyses described in this paper use phydms version 1.2.3.

I used phydms to infer a maximum-likelihood phylogenetic tree for each gene using GY94 with a single gene-wide *d* *N*/*d* *S* ratio (the M0 model in [4]). After fixing the tree topology to that estimated using GY94 M0, I re-optimized the branch lengths and model parameters by maximum likelihood for four additional models. The first is GY94 M3 [4], in which the likelihood for each site is a linear combination of those under three different *d* *N*/*d* *S* values, with these values and their weights shared across the whole alignment and optimized by maximum likelihood. The second is ExpCM. The third is ExpCM with the amino-acid preferences averaged across sites – this averaging makes the model non-site-specific, but captures any gene-wide trends in the deep mutational scanning data. The final is ExpCM with the amino-acid preferences randomized among sites – this model is still site-specific, but the site-specific parameters are no longer associated with the actual site for which they were measured.

I compared these models using Akaike Information Criteria (AIC) [43], which measures model fit penalized by the number of free parameters. Table 2 shows that ExpCM describe the evolution of all four genes far better than any other model. This table also shows that for *β*-lactamase, the new Stiffler et al [14] deep mutational scanning dataset informs ExpCM that are superior to those informed by the older Firnberg et al [34] deep mutational scanning dataset, although ExpCM informed by either dataset are vastly superior to any GY94 models. The huge superiority of ExpCM over the GY94 models is because ExpCM capture site-specific evolutionary constraints, as demonstrated by the fact that ExpCM in which preferences are averaged across sites are comparable to GY94. The poor performance of the randomized ExpCM is because a site-specific model only helps if the experimentally measured preferences are assigned to the correct sites. Indeed, Table 2 shows that randomly assigned site-specific preferences are so detrimental that they are nearly completely flattened by fitting a stringency parameter *β* that is close to zero, effectively making the randomized ExpCM non-site-specific. Overall, Table 2 confirms previous work [16, 17, 32, 33, 44] showing that experimentally informed site-specific models provide vastly improved descriptions of evolution.

**Table 2:** Site-specific ExpCM are vastly better than GY94 or ExpCM with preferences averaged or randomized across sites

Gene	Model	*Δ*AIC	Log likelihood	# free parameters: values of selection parameters
Gal4	ExpCM	0	–1048	6: *β*=0.82, *ω*=0.13
	GY94 M3	129	–1103	15: *ω*_1_=0.01, *ω*_2_=0.11, *ω*_3_=0.49, *p*_1_=0.50, *p*_2_=0.29
	GY94 M0	192	–1139	11: *ω*=0.06
	averaged ExpCM	196	–1146	6: *β*=1.07, *ω*=0.06
	randomized ExpCM	206	–1151	6: *β*=0.10, *ω*=0.07
*β*-lactamase	ExpCM	0	–3421	6: *β*=1.01, *ω*=1.02
	ExpCM (Firnberg data)	204	–3523	6: *β*=1.04, *ω*=0.65
	GY94 M3	564	–3694	15: *ω*_1_=0.07, *ω*_2_=0.55, *ω*_3_=6.24, *p*_1_=0.69, *p*_2_=0.17
	GY94 M0	765	–3798	11: *ω*=0.34
	averaged ExpCM	766	–3804	6: *β*=0.77, *ω*=0.35
	randomized ExpCM	790	–3816	6: *β*=0.10, *ω*=0.34
NP	ExpCM	0	–8624	6: *β*=2.43, *ω*=0.61
	GY94 M3	2175	–9703	15: *ω*_1_=0.00, *ω*_2_=0.16, *ω*_3_=1.31, *p*_1_=0.59, *p*_2_=0.24
	averaged ExpCM	2584	–9916	6: *β*=0.43, *ω*=0.11
	randomized ExpCM	2593	–9921	6: *β*=0.10, *ω*=0.11
	GY94 M0	2613	–9926	11: *ω*=0.11
HA	ExpCM	0	–7461	6: *β*=1.61, *ω*=0.60
	GY94 M3	1782	–8343	15: *ω*_1_=0.02, *ω*_2_=4.26, *ω*_3_=4.94, *p*_1_=0.59, *p*_2_=0.25
	averaged ExpCM	2137	–8530	6: *β*=0.42, *ω*=0.23
	randomized ExpCM	2157	–8539	6: *β*=0.10, *ω*=0.23
	GY94 M0	2176	–8544	11: *ω*=0.22

Another informative comparison is between the *d* *N*/*d* *S* of GY94 and the *ω* of ExpCM. ExpCM can represent protein-level constraint either via the site-specific amino-acid preferences or by shrinking *ω* to <1. In contrast, GY94 can only represent constraint by shrinking *d* *N*/*d* *S* even if the actual selection is for preferred amino acids at each site rather than against amino-acid change *per se* [45]. Table 2 shows that the ExpCM *ω* is always greater than the GY94 *d* *N*/*d* *S*. This effect is most striking for *β*-lactamase: while GY94 suggests selection against amino-acid change *per se* by fitting *d* *N*/*d* *S*=0.3, ExpCM indicate that this selection is actually accounted for by the site-specific amino-acid preferences by fitting *ω*=1. For the other three genes, the ExpCM *ω* is <1 indicating that the site-specific amino-acid preferences don’t capture all constraints, but the ExpCM *ω* is still always substantially greater than the GY94 *d* *N*/*d* *S*.

The ExpCM stringency parameter *β* also provides useful information. Recall that *β*>1 means that natural evolution selects for preferred amino acids with greater stringency than the deep mutational scanning. Table 2 shows that for both influenza genes (NP and HA), the stringency of natural selection exceeds that of the deep mutational scanning, indicating that the selection in experiments in [32] and [33] was not as rigorous as selection in nature. For *β*-lactamase, the stringency of natural evolution is approximately equal to that of the deep mutational scanning, providing a second indication (along with the fitting of *ω*≈1) that the experiments in [14] did an excellent job of capturing the constraints on *β*-lactamases in nature. Only for Gal4 is *β*<1: either the selections in [31] were more stringent than natural selection, or the measured preferences are not completely representative of those in nature and so *β* is fit to <1 to somewhat flatten these preferences.

**Fig. 2:**
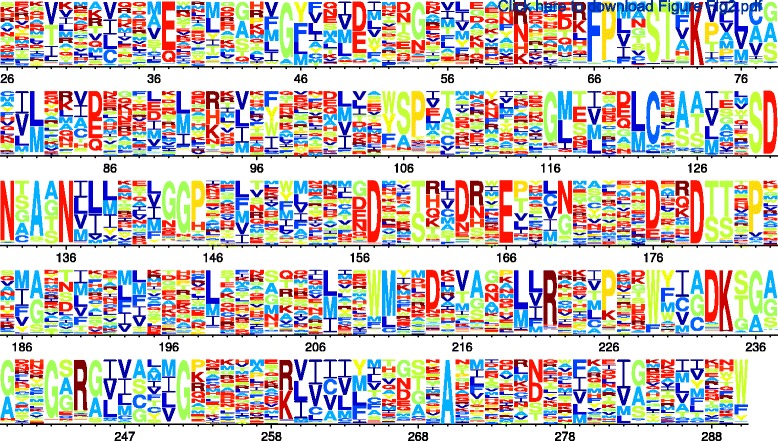
Site-specific amino-acid preferences for *β*-lactamase. The height of each letter is proportional to the preference for that amino acid at that site, and letters are colored by amino-acid hydrophobicity. These are the preferences experimentally measured in [14] for TEM-1 *β*-lactamase under selection with 2.5 mg/ml ampicillin, re-scaled by the stringency parameter *β*= 1.01 from Table 2. The re-scaling is done so that if the experimentally measured preference for amino-acid *a* at site *r* is *π*_*r*,*a*_, then the rescaled preference is proportional to (*π*_*r*,*a*_)^*β*^. The *β*-lactamase sequence is numbered using the Ambler scheme [82], meaning that residue numbers 239 and 253 are skipped. Comparable data for Gal4, NP, and HA are shown in Additional files 2, 3 and 4, respectively

The stringency-rescaled amino-acid preferences are in Fig. 2 and Additional files 2, 3 and 4. These figures reveal remarkable variation in constraint among sites, explaining why ExpCM better describe evolution than non-site-specific models. Overall, the results in this section verify that ExpCM offer a better evolutionary null model, and so motivate their use in identifying diversifying and differential selection.

### Experimentally informed site-specific models better detect diversifying selection

I used the ExpCM to identify sites of diversifying selection for amino-acid change. This was done by using phydms to fit *ω*_*r*_ and a synonymous rate for each site *r* via Eq. 5, fixing all other parameters at their optimized values. To compare to a standard *d* *N*/*d* *S* method, I also fit a *d* *N*/*d* *S* ratio and synonymous rate for each site using GY94 with all other parameters fixed to the values optimized under GY94 M3 (equivalent to the fixed effects likelihood or FEL method as implemented in [5]).

**Fig. 3:**
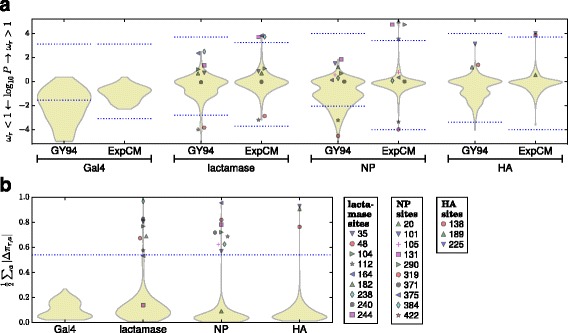
The experimentally informed models (ExpCM) identify many sites of diversifying or differential selection that are missed by a standard *d* *N*/*d* *S* analysis (GY94). **a** The violin plots show the distribution of *P*-values that a site is under diversifying selection for (positive numbers) or against (negative numbers) amino-acid change (*ω*_*r*_ indicates both the ExpCM parameter in Eq. 5 and the GY94 *d* *N*/*d* *S* ratio). The portion of the distribution above / below the dotted blue lines contains all sites for which there is support for rejecting the null hypothesis *ω*_*r*_=1 at a FDR of 0.05. When there are no sites with support at this FDR, the dotted blue lines indicate the *P*-value that would be needed for a site to have *ω*_*r*_>1 or <1 at a significance level of 0.05 using a Bonferroni correction. The *d* *N*/*d* *S* method identifies many sites of purifying selection, but fails to find any sites of selection for amino-acid change. The ExpCM model already accounts for basic functional constraints and so doesn’t identify any sites with *ω*_*r*_<1, but does identify sites of diversifying selection in all genes except Gal4 (which is not thought to evolve under pressure for phenotypic change). **b** The violin plots shown the distribution of differential selection at each site inferred with the ExpCM. Since Gal4 is not under selection for phenotypic change, I defined a heuristic threshold at 2-times the Gal4 maximum value of 0.27. At this threshold, sites of differential selection are identified for all three other genes. The legend labels all sites under diversifying or differential selection. This analysis was performed using phydms; Additional file 17 shows that similar results are obtained if the *d* *N*/*d* *S* analysis is instead performed using HyPhy [7]

Figure 3 a shows that ExpCM have much greater power to identify diversifying selection than the GY94 *d* *N*/*d* *S* method. For Gal4, GY94 finds many sites with *d* *N*/*d* *S*<1, but no sites with *d* *N*/*d* *S*>1 at an FDR of 0.05. As discussed in the Introduction, identifying sites with *d* *N*/*d* *S*<1 points to the naivety of the GY94 null model rather than unexpected biology, since any reasonable researcher would have already expected Gal4’s protein sequence to be under evolutionary constraint. The more plausible ExpCM null model finds that all sites in Gal4 are evolving as expected from the measurements in the lab (for no sites does it reject the null hypothesis *ω*_*r*_=1). For the other three genes, GY94 again finds that there are many sites with *d* *N*/*d* *S*<1 while failing to identify any sites with *d* *N*/*d* *S*>1 at an FDR of 0.05 – despite the fact that there is clear evidence that all three genes fix drug-resistance or immune-escape mutations. In contrast, the more realistic ExpCM find sites of diversifying selection for all three genes: there are three sites with *ω*_*r*_>1 in *β*-lactamase, four in NP, and two in HA.

To statistically validate the ExpCM approach for identifying diversifying selection, I used pyvolve [46] to simulate alignments of NP under ExpCM informed by the experimentally measured preferences and using the tree inferred for the actual NP sequences. In each simulation, I randomly selected five sites and placed them under diversifying with *ω*_*r*_ values ranging from 5 to 30. I then analyzed the simulated alignments for diversifying using the ExpCM and the FEL-like GY94 *d* *N*/*d* *S* method. As shown in Additional file 5, ExpCM consistently outperformed GY94 at identifying the simulated sites of diversifying selection. Additional file 5 also shows that the Benjamini-Hochberg procedure [28] effectively controlled the false discovery rate. These simulations demonstrate the statistical soundness of the ExpCM approach for identifying diversifying selection.

Both the FEL-like GY94 *d* *N*/*d* *S* method and the ExpCM used for the analysis in Fig. 3 a test for diversifying selection across the phylogeny. But in many cases, diversifying selection is episodic. Therefore, *d* *N*/*d* *S* methods have been extended to identify sites under diversifying selection in only some lineages [6, 47–49]. I used one of these methods, MEME [6], to test for episodic diversifying selection. Additional file 6 shows that MEME identifies one site of diversifying selection each in *β*-lactamase and NP, and no sites in HA or Gal4. This makes MEME more powerful than the FEL-like GY94 method but still less powerful than ExpCM. However, MEME and ExpCM outperform the FEL-like GY94 method for orthogonal reasons: MEME is superior because it can identify episodic selection, whereas ExpCM are superior because they account for functional constraints on individual sites. In principle, it should be possible to merge ExpCM with methods to identify episodic diversifying selection.

A variety of other *d* *N*/*d* *S* methods have also been developed. The most prominent other class includes so-called “random effects” methods that use an empirical Bayesian approach to share information about the distribution of *d* *N*/*d* *S* across sites [2, 50–52]. The relative pros and cons of “random effects” methods versus the “fixed effects” methods used in this paper remain an area of active discussion [5, 53]. It is beyond the scope of the current study to compare these two classes of methods. Here I simply note that as with the test for episodic selection described in the previous paragraph, ExpCM substitution models could in principle also be incorporated into the “random effects” framework, since the essential differences between “random effects” and “fixed effects” methods are due to how parameters are handled rather than the substitution model itself.

Overall, the results in this section show that ExpCM are better at identifying diversifying selection than several standard *d* *N*/*d* *S* methods. The reason for this superiority is that the ExpCM account for variation in the inherent constraints on different sites, and so have greater power to recognize when a functionally constrained site is changing more rapidly than expected.

### Experimentally informed site-specific models enable detection of differential selection

ExpCM also enable identification of differential selection for unexpected amino acids. I used phydms to estimate the differential preference *Δ* *π*_*r*,*a*_ of each site *r* for each amino-acid *a* by optimizing the product of Eq. 6 and Eq. 7 after fixing all other parameters. The differential selection at each site *r* was quantified as $\frac {1}{2} \sum _{a} \left |\Delta \pi _{r,a}\right |$, which can range from zero to one.

Figure 3 b shows the distribution of site-specific differential selection. As expected, no sites in Gal4 are under strong differential selection. But for each of the other genes, a small subset of sites are under strong differential selection. I heuristically classified differential selection as “significant” if it exceeded 2-times the maximum value for Gal4. At this threshold, there are seven sites of differential selection in *β*-lactamase, nine in NP, and three in HA. So overall, Fig. 3 b suggests that most sites are evolving as expected in all four genes, but a small fraction of sites are under differential selection in *β*-lactamase, NP, and HA due to their roles in drug resistance or immune escape. This result is concordant from what we expect given biological knowledge about the selection pressures on these genes. Note that similarly reasonable results are *not* obtained using the non-phylogenetic Kullback-Leibler divergence to measure differences between amino-acid frequencies in nature and the experimentally measured amino-acid preferences (Additional file 7). This fact emphasizes the importance of examining evidence for diversifying selection in a phylogenetic framework rather than analyzing them using statistical approaches that treat them as independent samples from some underlying ensemble.

**Fig. 4:**
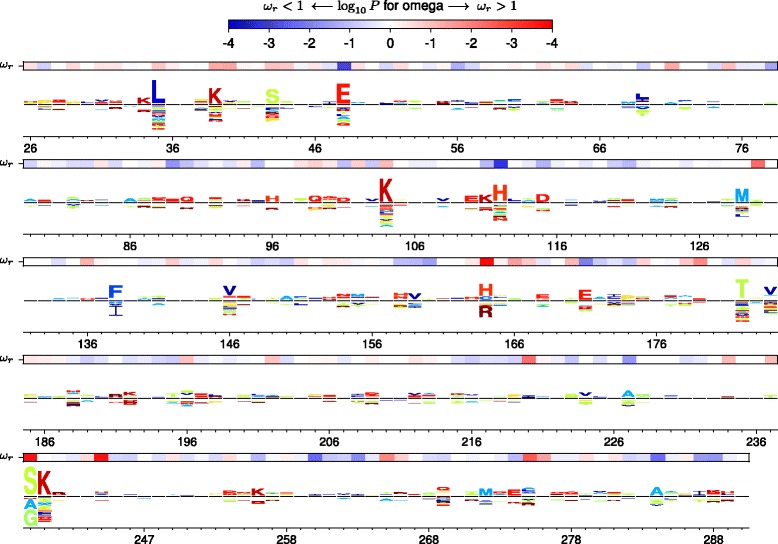
Site-specific selection on *β*-lactamase inferred with experimentally informed models. The height of each letter above/below the black center line is proportional to the differential selection for/against that amino acid at that site relative to what is expected from the amino-acid preferences in Fig. 2. The overlay bar shows the evidence for diversifying selection at each site, which is manifested by strong evidence for a ratio *ω*_*r*_ of nonsynonymous to synonymous substitution rates that is higher (*red*) or lower (*blue*) than expected from the amino-acid preferences. The *β*-lactamase sequence is numbered using the Ambler scheme [82], meaning that residue numbers 239 and 253 are skipped. Comparable data for Gal4, NP, and HA are shown in Additional files 8, 9, and 10, respectively

A more detailed portrayal of the diversifying selection at each site is in Fig. 4 and Additional files 8, 9, and 10. For each site, these images display the evidence for diversifying selection, the strength of differential selection, and the differential preference for each amino acid at sites under non-negligible differential selection.

There are sites in *β*-lactamase, NP, and HA that are under both diversifying and differential selection, but there are also sites that are only under one of these forms of selection (Fig. 3). These findings make sense: often, pressure for amino-acid change will drive multiple substitutions to non-preferred amino-acid identities, leaving traces of both types of selection. But sometimes, a relatively unconstrained site substitutes to a variety of different amino acids, leading to diversifying but not differential selection. In other cases, a site fixes just one or a few substitutions to a non-preferred amino acid that confers some enduring phenotypic benefit, leading to differential but not diversifying selection.

### The identified sites of selection are consistent with existing biological knowledge

The ExpCM identified sites of differential and diversifying selection in all three genes that are undergoing adaptive evolution (*β*-lactamase, NP, and HA), while GY94 identified no sites with *d* *N*/*d* *S*<1 in any of the genes. But before concluding that this result indicates the superiority of the ExpCM, we must answer the following question: are the identified sites actually the locations of substitutions that have altered evolutionarily relevant phenotypes? To answer this question, I examined the literature on drug resistance in *β*-lactamases and immune escape by NP and HA (Table 3).

**Table 3:** At most sites of selection identified using ExpCM, mutations affect drug resistance or immune escape

Gene	Site	Affects biologically relevant phenotype?
*β*-lactamase	35	No evidence implicating this site in resistance [15]
	48	No evidence implicating this site in resistance [15]
	104	E104K involved in *β*-lactam resistance [15]
	112	No evidence implicating this site in resistance [15]
	164	R164C, R164H, and R164S involved in *β*-lactam resistance [15]
	182	M182T potentiates resistance [15]
	238	G238S involved in *β*-lactam resistance [15]
	240	E240K involved in *β*-lactam resistance [15]
	244	R244C, R244H, and R244S involved in inhibitor resistance [15]
NP	20	In a T-cell epitope [94]
	101	In an antibody epitope [95]
	105	In a T-cell epitope [96]
	131	Not part of known immune epitope
	290	In an antibody epitope [97]
	319	Not part of known immune epitope
	371	In an antibody epitope [98, 99]
	375	E375G interacts with T-cell escape mutation at 384 [58, 100]
	384	R384G and R384K are T-cell escape mutations [38, 100]
	422	K422R is a T-cell escape mutation [101]
HA	138	Contacts antigenic-site residues defined by the experiments of [40]
	189	Contacts antigenic-site residues defined by the experiments of [40]
	225	An antigenic site residue defined by the experiments of [40]; also affects receptor-binding specificity and so known to undergo substitutions both during host adaptation and viral passaging in the lab [84–87]

For *β*-lactamases, [15] reports 18 sites at which mutations known to affect resistance are observed in clinical isolates. The ExpCM identify 9 sites of selection; 6 of these 9 sites are among the 18 known sites of resistance mutations (Table 3). There are 263 residues in the mature *β*-lactamase protein, so we can reject the possibility that the identified sites are not associated with resistance mutations (*P*=10^−6^, Fisher’s exact test). So for *β*-lactamase, the ExpCM mostly identify sites that have been independently shown to affect drug resistance.

NP is under immune selection to escape T cells [10, 38] and probably also antibodies [54, 55]. The ExpCM identify 10 sites of selection. I searched the literature and found reports that 8 of these 10 sites are relevant to immune escape (Table 3). So for NP, the ExpCM mostly identify sites that have been independently shown to affect immunogenicity.

HA is under immune selection to escape antibodies. Caton et al [40] used antibodies to map escape mutations in H1 HA. A reasonable definition of the antigenic portion of HA is the set of sites identified in [40] plus any sites in three-dimensional contact with these sites (a contact is defined as a *C*_*α*_−*C*_*α*_ distance ≤6Å in PDB 1RVX). Using this definition, 86 of the 509 sites in the HA ectodomain are in the antigenic portion of the molecule. The ExpCM identify 3 sites of selection, all of which are in the antigenic portion of HA. We can reject the possibility that these identified sites are not associated with the antigenic portion of the molecule (*P*=0.005, Fisher’s exact test). So for HA, the ExpCM identify sites that have been independently shown to affect immunogenicity.

**Fig. 5:**
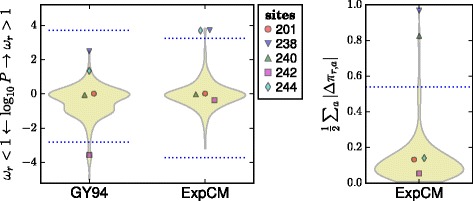
The experimentally informed models (ExpCM) correctly identify the three *β*-lactamase sites in Fig. 1 that contribute to drug resistance. Figure 1 showed five sites in *β*-lactamase, three of which (238, 240, and 244) experience substitutions that contribute to drug resistance. However, a *d* *N*/*d* *S* analysis (GY94) fails to identify any of these sites as under diversifying selection (*d* *N*/*d* *S*>1) at a FDR of 0.05 for testing all sites (*dotted blue lines*). In contrast, ExpCM correctly determine that the three resistance sites are under diversifying (238 and 244) or differential (238 and 240) selection, and that the two non-resistance sites (201 and 242) are evolving as expected. ExpCM outperform the *d* *N*/*d* *S* method because they implement a null model that accounts for the site-specific amino-acid preferences shown in Fig. 1; for instance, this null model is not surprised that site 242 remains fixed at the highly preferred amino-acid R, but does find it noteworthy that site 240 substitutes to K multiple times even though that is not a particularly preferred amino acid

Overall, these results show that sites of selection identified by ExpCM are indeed the locations of substitutions that alter evolutionarily relevant phenotypes. For a concrete illustration of sites of adaptive substitutions that are identified by ExpCM but not by a *d* *N*/*d* *S* method, Fig. 5 shows the results of the ExpCM analysis of the five example sites in *β*-lactamase discussed in the Introduction and Fig. 1. Three of these five sites experience substitutions that affect resistance, but a *d* *N*/*d* *S* method fails to flag any of them as under diversifying selection (*d* *N*/*d* *S*>1) since it doesn’t account for site-specific constraints (Fig. 1). Figure 5 shows that ExpCM correctly identify all three resistance sites as under diversifying or differential selection, while finding that the non-resistance sites are evolving as expected. Visual inspection of the two figures provides an intuitive explanation of why accounting for site-specific amino-acid preferences makes ExpCM so much more powerful at identifying sites of selection to alter evolutionarily relevant phenotypes.

## Discussion

I have described an approach that uses experimentally informed models to identify sites of biologically interesting selection in protein-coding genes. This approach asks the following question: *Is a site evolving differently in nature than expected from constraints measured in the lab?* In contrast, traditional *d* *N*/*d* *S* methods simply ask: *Is a site evolving non-neutrally?* The former question is sometimes more informative than the latter. It is by now abundantly clear that most protein residues are under some type of constraint, so finding that a site evolves non-neutrally is often unsurprising. Instead, we want to identify sites of substitutions that have altered evolutionarily relevant phenotypes. As demonstrated here, experimentally informed models have much greater power to identify such sites. The improvement is remarkable: while a *d* *N*/*d* *S* method fails to find any sites of adaptive evolution in the genes examined, experimentally informed models identify 22 sites of diversifying or differential selection, most of which fix mutations that have been independently shown to affect drug resistance or immunogenicity.

What accounts for the improved power of the experimentally informed site-specific models? As vividly illustrated by the deep mutational scanning studies that provide the data used here (Fig. 2 and Additional files 2, 3, and 4), there is vast variation in the constraints on sites within a protein. Therefore, the significance that we should ascribe to a substitution depends on where it occurs: several changes at an unconstrained site may be unremarkable, but a single substitution away from a preferred amino acid at a constrained site probably reflects some powerful selective force. Whereas *d* *N*/*d* *S* methods treat all substitutions equally, the models used here evaluate the significance of each substitution in the context of the experimentally measured amino-acid preferences of the site at which it occurs.

Does this reliance on experimental measurements make the approach less objective? At first glance, the fact that *d* *N*/*d* *S* methods are uncontaminated by messy experiments feels reassuring. In contrast, experimentally informed models are dependent on all the subjective decisions associated with experimental design and interpretation. In addition, experiments in the lab may fail to fully capture all the selection pressures operating in nature. But in truth, experimentally informed models simply make explicit something that is already true: we define positive selection with respect to a null model for evolution in the absence of this selection. At least for the genes examined here, sites of known adaptive mutations are better identified by leveraging imperfect experiments that capture many of the constraints on natural evolution than by objectively testing the implausible null hypothesis that every site is evolving neutrally.

An assumption of experimentally informed site-specific models is that amino-acid preferences are conserved among the homologs under analysis. At first glance this assumption seems tenuous – epistasis can shift the effects of mutations as a gene evolves [56–58]. But it is rare for epistatic shifts to be large enough to undermine the advantage of site-specific models: this fact is demonstrated by direct experiments [32, 59, 60], the observation that parallel viral lineages tend to substitute to the same preferred amino acids at each site [61], and the empirical superiority of site-specific models in fitting phylogenies of diverged homologs (Table 2, [17, 32]). Therefore, epistasis does not subvert the basic advantage of a model informed by site-specific amino-acid preferences.

Of course, experimentally informed site-specific models require measurement of amino-acid preferences. However, advances in deep mutational scanning will make this requirement less and less of an impediment [11, 12]. In a fitting twist, one of the pioneers of deep mutational scanning [11] was also the first to sequence a gene from influenza [62, 63]. At the time, sequencing the homologous gene from thousands of other viral strains must have seemed unimaginable – a few decades later, for this study I had to subsample the ≫10^5^ publicly available influenza sequences down to a manageable number. The core techniques of deep mutational scanning – sequencing and gene/genome engineering – are improving at a similar pace, so coming years will see measurement of the amino-acid preferences of many more genes.

Another possibility is to use non-experimental strategies to inform site-specific models like the one here. One strategy is to predict site-specific constraints from higher-level properties such as solvent accessibility [64–66] or via molecular simulation [67–70]. It remains unclear whether such non-experimental strategies can predict site-specific amino-acid preferences with sufficient accuracy to inform substitution models that can match the ExpCM used here. Another strategy is to infer preferences from naturally occurring sequences [30, 71–75]. If care is taken to avoid the over-fitting that could accompany inferring preferences from the same naturally occurring sequences that are being analyzed for selection, then this might be a viable approach. Indeed, while the current paper was under review, Rodrigue and Lartillot published an elegant study that implements an approach along these lines [76]. But I suggest that direct measurement of amino-acid preferences via deep mutational scanning may well prove the best solution in many cases: after all, biology is full of properties that are challenging to predict or infer, but are now routinely measured in high-throughput.

Overall, I have described a new approach that leverages high-throughput experimental data to identify sites of selection in protein-coding genes. This approach clearly outperforms a standard implementation of the widely used *d* *N*/*d* *S* strategy, however there is much room for improvement. The utility of the *d* *N*/*d* *S* strategy has been enhanced by innovations that have made it possible to do things like test for selection only along certain branches [6, 49], utilize Bayesian approaches to share information across sites [2, 50–52], better incorporate synonymous rate variation [77], and more rapidly perform the computational analyses [52, 78]. Most of these innovations could also be used in combination with the experimentally informed models described here. Methodological improvements of this sort, coupled with growing amounts of deep mutational scanning data, could make experimentally informed models an increasingly powerful tool to identify genotypic changes that have altered phenotypes of interest.

## Methods

### Software implementing the analyses

The algorithms described in this paper are implemented in the phydms software package, which is available at https://github.com/jbloomlab/phydms. This package is written in Python, and uses cython to interface with and extend Bio++ (http://biopp.univ-montp2.fr/, [41, 42]) for the likelihood calculations. Special thanks to Laurent Guéguen and Julien Dutheil for generously making the cutting-edge version of Bio++ available and providing assistance in its use. The software uses dms_tools (https://github.com/jbloomlab/dms_tools, [13]) and weblogo (http://weblogo.threeplusone.com/, [79]) for visualizing the results. The analyses in this paper used phydms version 1.2.3.

### Amino-acid preferences for the four proteins

The amino-acid preferences were taken from previously published deep mutational scanning experiments. For NP, the preferences were taken from [32], using the average of the measurements for the two NP variants. For HA, the preferences were taken from [33]. For *β*-lactamase, [14] provides “relative fitness” scores, which are log10 enrichment ratios. I used the scores for the selections on 2.5 mg/ml of ampicillin (the highest concentration), averaging the scores for the two replicates. Following the definition in [13] of the preferences as the normalized enrichment ratios, the preferences *π*_*r*,*a*_ are calculated from the relative fitness scores *S*_*r*,*a*_ so that $\pi _{r,a} \propto \max \left (10^{S_{r,a}}, 10^{-4}\right)$ and $1 = \sum _{a} \pi _{r,a}$. For Gal4, [31] provides “effect scores”, which are the log2 of the enrichment ratios. The preferences are calculated from the effect scores *E*_*r*,*a*_ so that $\pi _{r,a} \propto \max \left (2^{E_{r,a}}, 2 \times 10^{-4}\right)$ and $1 = \sum _{a} \pi _{r,a}$. A few effect scores are missing from [31], so these scores are set to the average for all mutations for which scores are provided. The formulas to convert the *β*-lactamase and Gal4 scores to preferences include the max operators to avoid estimating preferences of zero; the minimal allowable values specified by the second argument to these operators are my guess of the lowest frequency that would have been reliably observed in each experiment.

For the comparison of the two different deep mutational scanning datasets for *β*-lactamase shown in Table 2, the measurements from the Firnberg et al [34] deep mutational scanning were converted into site-specific amino-acid preferences as described in [17].

### Alignments of naturally occurring sequences for each protein

For NP, the sequence alignment was constructed by extracting all post-1950 full-length NPs in the Influenza Virus Resource [80] that are descended in purely human lineages from the 1918 virus (H1N1 from 1950–1957 and 1977–2008, H2N2 from 1957–1968, and H3N2 from 1968–2015), and retaining just two sequences per-subtype per-year to yield a manageable alignment. The rationale for using only post-1950 sequences is that most viruses isolated before then were passaged extensively in the lab prior to sequencing. For HA, the alignment was constructed by extracting all post-1950 sequences in the human seasonal H1N1 lineage (H1N1 from 1950–1957 and 1977–2008), and retaining just four sequences per year to yield a manageable alignment. For *β*-lactamase, the alignment consists of the TEM and SHV *β*-lactamases used in [17]. For Gal4, a set of homologs was obtained by performing a tblastn search of the Gal4 DNA-binding domain used by [31] against wgs (limiting by saccharomyceta (taxid:716545)) and chromosomes for hits with *E*≤0.01, and retaining only sequences that aligned to the Gal4 DNA-binding domain with ≥ 70% protein identity and ≤ 5% gaps. For all genes, alignments were made pairwise to the sequence used for the deep mutational scanning with EMBOSS needle [81], and sites were purged if they were gapped in that sequence.

### Sequence numbering

In the figures and tables, the residues in NP are numbered sequentially beginning with one at the N-terminal methionine. The residues in HA are numbered using the H3 numbering scheme (the one used in PDB 4HMG), and the site-specific selection analysis is performed only for the residues in HA ectodomain (residues present in PDB 4HMG). The residues in *β*-lactamase are numbered using the Ambler scheme [82]. The residues in Gal4 are numbered using the scheme in [31].

### Data availability

The software package that implements the algorithms described in this paper is available at https://github.com/jbloomlab/phydms. The analyses were performed using version 1.2.3 of the phydms software. Data and scripts to perform the specific analyses are provided as Additional files 11, 12, 13, 14 and 15.

## Reviewers’ comments

### Reviewer Report 1: Sebastian Maurer-Stroh, Bioinformatics Institute (BII), A*STAR, Singapore

Reviewer summary –

Interesting well conceived approach.

Author response: *Thank you for the kind words.*

Reviewer recommendations to author –

This is an interesting approach to overcome simplifications of dN/dS site selection models by using site-specific experimental data from deep mutational scanning. As beautifully detailed and desirable this sounds, one should not forget that the experimental setup is detrimental for the types or aspects of protein function that can actually be investigated which directly influences the range of obtainable interpretations. For example, influenza hemagglutinin has multiple roles to fulfill on top of antigenic drift such as pH-dependent conformational changes and receptor binding. Similarly, functional roles of the nucleoprotein are not only thermal stability and immune response evasion but also RNA packing and sub-cellular shuttling. Also beta-lactamases will mutate differently under different pressures from different antibiotics or in competition with other bacteria. The difficulty of the experimental setup to represent the full complexity of natural selection pressures is not always just a limiting factor but looking only at some aspects of function at any one time allows elegantly gauging details of specifically targeted evolutionary forces at play. The notion of the critical influence of the experimental setup is mentioned in the discussion but would be good to be included also in the introduction.

Author response: *This is an important point. I have elaborated the paragraph in the Discussion that describes how experiments in the lab will sometimes fail to fully capture selection in nature (This is the paragraph beginning, “Does this reliance on experimental measurements make the approach less objective?” I have also added mention of this point in the Introduction by emphasizing that “lab measurements are undoubtedly imperfect proxies for actual selective constraints in nature.”*

*The reviewer also makes excellent points regarding influenza hemagglutinin in particular. Although I do not go into these points in the current manuscript (which focuses more on the general approach than the details of HA), the reviewer’s intuition is validated by recent work for my group specifically focusing on HA [* 44 *] which found that the experimentally informed models identify both sites of actual positive selection from immunity and sites subject to lab-specific selection pressures related to proteolytic activation of HA. However, despite these caveats, I think that the current manuscript clearly demonstrates that site-specific models informed by imperfect experiments are superior to the much more unrealistic standard non-site-specific models.*

The formalism of the approach is well developed and intuitively makes sense but the practical result for hemagglutinin left me a bit wanting. Certainly the identified sites for HA in Table 3 are important but they seem only a small subset of such sites that can be identified with other methods (e.g. SLAC from HyPhy package over naturally occurring sequences finds dozens that can be rationalized to make sense through overlap with known epitopes etc). Could it simply be that the, in some cases, used heuristic Gal4-based thresholding is too conservative and considering less stringent criteria would find more of the presumably true sites?

Author response: *I think the relative paucity of sites identified in HA is due to the fact that the analysis focuses on seasonal H1 HA rather than H3 HA. For instance, I ran the H1 HA alignment used in this paper through* SLAC *as implemented in the* DataMonkey *web interface to* HyPhy *(data not shown). The* SLAC *analysis only identified two sites of positive selection for the H1 alignment. I would expect that all approaches would identify more sites in H3 HA, since human H3N2 influenza undergoes more rapid antigenic drift than human seasonal H1N1 influenza [* 83 *]. Such an analysis will be possible once deep mutational scanning data are available for an H3 HA.*

By the way, the criterion of Caton epitope residues plus everything within 6A does includes a lot of structurally buried residues. Maybe an additional surface accessibility criterion to enrich for direct epitope candidates may be justifiable here? If I am not wrong, HA 225 (in H3 numbering in Table 3) is a classical host/passage specificity position in H1 context and it is good to be highlighted by the new approach but its potentially broader functional importance on receptor binding should also be mentioned and referenced accordingly.

Author response: *These are both good points. The three sites of selection listed in the table are at least partially surface-exposed. As the reviewer points out, some of the 89 sites within 6Å are buried, and so are probably not true antigenic sites. Accounting for this fact would deflate the denominator in the Fisher’s exact test that we use to test the significance that we are identifying true antigenic sites, and so further improve the P-value for supporting the validity of our ExpCM method. However, I prefer to be conservative and keep all 89 sites in the denominator, since in some cases mutations at buried sites may still introduce slight conformational changes or N-linked glycosylation motifs that escape antibodies*.

*The point about HA site 225 in receptor-binding is a good one. I have added a line in the table that emphasizes that mutations at site 225 are implicated in both host adaptation and lab passaging adaptation via changing receptor binding, and have cited the following relevant references: [* 84 *–* 87 *]*.

The following additional points are meant to stimulate further thoughts for future work: Empirical average (neither site- nor protein-specific) amino acid substitution tables have been derived en masse since the early works of Dayhoff (PAM, JTT, BLOSUM,...). Picking one of the most popular, BLOSUM62, how similar or different is it for the studied proteins’ ExpCM results?

Author response: *Good question. Empirical amino-acid substitution matrices themselves cannot be directly substituted for codon substitution models. But there are a variety of empirical codon substitution models, which combine empirical amino-acid substitution models with codon substitution models. One such set of models are Kosiol et al 2007 models [* 88 *]. In prior work [* 16 *,* 17 *,* 33 *] I have compared these Kosiol 2007 models to the various forms of the Goldman-Yang style models used here, as well as earlier versions of the ExpCM. As described in that prior work, the Kosiol 2007 models in general were not substantially better (and were often actually worse) than the Goldman-Yang models in terms of phylogenetic fit. Therefore, it appears that an empirical model that tries to account for amino-acid substitutions in a way that is NOT site-specific does not lead to substantial improvements. This is probably because protein-level constraints are highly site-specific, and cannot effectively be modeled in an “average” across sites.*

Classical substitution matrices are traditionally derived from globular regions of proteins forming 3D structures but un- or dynamically structured N- or C-terminal stretches are also under selection pressure for targeting motifs and other constraints. An unbiased but complete scanning method may be equally applicable also in non-globular regions and pinpoint critical sites often neglected by earlier approaches?

Author response: *This is another good question. As the reviewer suggests, I would expect that perhaps the site-specific amino-acid preferences for unstructured protein domains to be quite a bit different than for globular proteins. To my knowledge, no one has yet performed deep mutational scanning on an unstructured protein domain. But once such experiments are done, as the reviewer suggests, it would be very interesting to test whether such experiments could inform substitution models.*

On the complexity of adaptive mutations in the substrate binding pocket of beta-lactamases, I found it curious that antibiotics resistance genes in microbiomes of an un-contacted Amazonian tribe had the capacity to also neutralize synthetic man-made antibiotics they have never been exposed to (http://www.sciencemag.org/news/2015/04/resistance-antibiotics-found-isolated-amazonian-tribe). This highlights plasticity of the natural repertoire of substrate binding pocket residues to accommodate a broad range of unknown substrates directly or with few mutations.

Author response: *This is an interesting observation. As more deep mutational scanning data sets become available, it will be interesting to compare the inherent plasticity of different active sites.*

Adaptive mutations are of great importance not just in the context of pathogens but it would be interesting to also apply deep scanning and ExpCM on key genes in human diseases (P53, KRas, EGFR, …)

Author response: *This is a great suggestion. Some recent studies by other groups have already started to move in this direction; see for instance [* 89 *,* 90 *]. These studies may have the potential to aid in the prospective identification of disease-causing human mutations.*

Last but not least, the manuscript and suppl. material with code links are commendably complete descriptions of the work.

Author response: *Thanks! Hopefully the availability of the code and data will help enable others to extend and improve the approaches described in this manuscript.*

Additional responses from reviewer after reading the revised version. The quoted text indicates the author’s comments in the revision:

“I think the relative paucity of sites identified in HA is due to the fact that the analysis focuses on seasonal H1 HA rather than H3 HA.” Indeed, could be true.

“... keep all 89 sites in the denominator, since in some cases mutations at buried sites may still introduce slight conformational changes or N-linked glycosylation motifs that escape antibodies.” Ok to keep all 89 sites for this paper but remove in the response the comment on buried N-glycosylation sites. The latter most commonly are not buried due to the simple necessity of access for the modifying enzyme machinery [91].

Author response: *The reviewer is correct that the N-linked glycans themselves are not buried. I had meant that in some cases the Ser/Thr in the Asn-Xaa-Ser/Thr glycosylation motif might be buried, but admittedly this is probably a rare event.*

“As described in that prior work, the Kosiol 2007 models in general were not substantially better (and were often actually worse)... ” Sure, I did not mean that they would be better in performance but more that it might be interesting to study trends in observed differences to possibly improve them with some extra rules e.g. something that would filter out less reliable sites where differences are always high. In other words, some amino acid substitution pairs may be more site-specific than others? In any case, partially addressed before and possible extension for future work.

Author response: *I agree that this is an interesting area for future work.*

“To my knowledge, no one has yet performed deep mutational scanning on an unstructured protein domain. But once such experiments are done, as the reviewer suggests, it would be very interesting to test whether such experiments could inform substitution models.” Most proteins are not fully structured but typically feature flexible N- and C-termini as well as often only partially structured longer loop regions. One way to define these unstructured regions is by looking for unresolved residues in crystal structures despite being part of the used sequence. These are easy to see when looking at the sequence tab of PDB files online. In fact for the H1N1 HA deep scan, it seem the author has deep scanning data for ∼18 unstructured residues in the N-terminus and 60 in the C-terminus [44]. Surprisingly there seems to be quite some constrained sites in the C-term here which also may point to functional importance as motifs or partial or conditional structure.

Author response: *This is a good idea – it would be interesting to specifically look at unstructured regions in proteins that have already been studied by deep mutational scanning. Such an analysis is beyond the scope of the current study, but is an interesting topic for future work. As the reviewer notes, the conservation at some sites in the C-terminus of HA is compatible with the fact that parts of the transmembrane domain and cytoplasmic tail are important for virion formation, such as via interactions between HA’s cytoplasmic tail and the matrix protein.*

### Reviewer Report 2: Olivier Tenaillon, INSERM, France

Reviewer summary –

In his manuscript entitled “Identification of positive selection in genes is greatly improved by using experimentally informed site specific models”, Jesse Bloom propose to use quantitative information based in deep mutational scanning experiments to detect selection in phylogenies. In previous articles, he proposed to use such information to improve the phylogenetic reconstruction, in the present one he extends the approach to detection of selection, the rational being that a better underlying model allows a finer detection of selection, and a site specific model gives more power to detect local effects. He applies his method to 4 genes, one in which no selection is expected and 3 in which there are target sites for selection. The results suggest a better detection of sites under selection. I really appreciated the approach used and have just minor comments.

Author response: *Thank you for the nice summary and kind words about the manuscript.*

Reviewer recommendations to author –

The method relies on the use of deep mutational scanning experiments, but does not mention how good and precise these experiments have to be. For instance, the Stiffler et al experiments [14] on beta-lactamases are done after 3 generations of growth and give mostly a growth, no growth information (actually Firnberg and Ostermeier data [34] would have been more appropriate as they provide a much finer resolution). Indeed, in that paper the distribution of fitness is almost completely bimodal for mutation effects. These experiments are much less costly than others that will do deep scanning with much more time points (or concentrations) and therefore with higher fitness resolution for the mutants. So how important is the precision of the experimental data? Would a binary fit for each amino acid mutation work as well? This is important for two reasons: first it can define somehow that price required to get a good signal with mutational scanning. Second, if the data are binary, then mutation prediction approach may be relevant. In a recent paper, Figliuzzi et al (MBE, 2016 [75] that should at least be cited along side with Hopf in *arRxiv* [74]), Martin Weigt’s group showed that the DCA and Independent model based on protein alignment were providing a good prediction of mutation effects produced in experiments especially on grow no-grow kind of data. If the improvement of the present approach is not very sensitive to the quality of the experimental data, then it would gain incredibly in usage if predictions from pfam alignment such as the ones done by DCA were used rather than costly experiments.

Author response: *These are all great points*.

*The first question is how to choose the which deep mutational scanning dataset to use to inform the substitution models. As the reviewer points out, there are currently two deep mutational scanning datasets for beta-lactamase: the one by Stiffler et al used in the current manuscript [* 14 *], and an earlier dataset by Firnberg and Ostermeier [* 34 *]. In prior work [* 17 *], I have shown that the Firnberg dataset also improves phylogenetic fit. But the initial version of this manuscript only used the newer Stiffler data set. So how do we know which is better? We can compare how well different deep mutational scanning datasets actually describe the constraints on natural evolution using maximum-likelihood phylogenetics via AIC, exactly as is traditionally done to compare substitution models [* 43 *]. Specifically, we can perform phylogenetic fitting of ExpCM informed by each dataset to see which one yields a higher likelihood of the actual natural sequences. The new Table* 2 *now includes analyses with ExpCM informed by each deep mutational scanning dataset. As can be seen from this table, ExpCM informed by the Stiffler dataset describe the natural evolution of* *β* *-lactamase better than ExpCM informed by the Firnberg dataset (* *Δ* *A* *I* *C* *= 204). Therefore, by the criterium typically used to compare substitution models, the Stiffler dataset is superior. Note however that either dataset informs ExpCM that are clearly better than standard GY94-type models*.

*The foregoing analyses do not provide a basis for concluding why the Stiffler dataset is superior to the Firnberg one. As the reviewer notes, one difference is that the more extended selection in the Stiffler et al experiments leads to more binary measurements. But the differences could also be due to reasons that are more technical than biological. For instance, Stiffler et al perform two full biological replicates of their deep mutational scanning, and I have used the average of the two replicates – this averaging presumably reduces experimental noise. In contrast, Firnberg et al did not perform replicates of their experiment, so perhaps there is more noise that has not been averaged away. Consistent with this idea, analyses of other genes have shown that averaging across experimental replicates of deep mutational scanning typically improves ExpCM [* 44 *], presumably by reducing the effects of measurement noise*.

*Thanks for pointing out the Figliuzzi et al [* 75 *] study that predicts mutational effects from sequence alignments. I have added mention of this study to the paragraph in the Discussion that addresses whether site-specific amino-acid preferences could be computationally inferred from natural alignments rather than measured experimentally (this is the paragraph beginning “Another possibility is to use non-experimental strategies to inform site-specific models like the one here.”). The short answer is that I do not know whether computational methods like those used in Figliuzzi et al [* 75 *] could be used in place of deep mutational scanning – but certainly I agree that this would greatly expand the utility of approaches like the one that I describe in the current manuscript. One caveat about inferring the preferences from natural sequence alignments is that care must be taken to avoid over-fitting the data, as the preferences would then come from the same alignment that is being analyzed phylogenetically – in my current manuscript, the preferences are from a separate dataset (the deep mutational scanning) from the natural sequence alignment. However, it may be possible to infer the preferences without overfitting – see for instance a paper by Rodrigue and Lartillot [* 76 *] that was published while the current manuscript was under review. Certainly I hope that the current manuscript will help inspire future work to see if the site-specific amino-acid preferences can also be obtained in other “cheaper” ways than deep mutational scanning – although I would note that deep mutational scanning itself is also getting progressively cheaper.*

The differential selection is interesting but not as intuitive than the diversifying one. The experiments being made in the lab, they may lack some facets of selection. So the test will tell us if sites are significantly different from the selection in the lab. However, we can not, in many cases, know whether this is a true mark of selection in the wild or a limited power of the experimental setting to provide a good model.

Author response: *This is a good point. I have added text to the Discussion that emphasizes that the diversifying selection test looks for differences between selection in nature and what is expected given measurements in the lab. I have emphasized why this will sometimes (but not always) be informative for identifying mutations of biological interest.*

In the different sets of genes studied here the difference of selection between laboratory and other experiments is relevant: lack of immune system, or lack of new antibiotic, but how general can that be? It could be worth discussing briefly that issue, to give some intuition to future users about the meaning of the signal they may get.

*This is a good point. I have added text that describes how the tests are especially useful when we know that there are selection pressures (such as immunity or drug resistance) that are present in nature but not in the lab. Similar situations where there are known external pressures in nature but not in the lab will occur sometimes (as in the case of the influenza genes and* *β* *-lactamase), but not in other cases (such as Gal4).*

It would be appropriate to plot the trees of each gene alignment that are used for inference and present the state of the candidate mutations.

*Given the large number of candidate mutations, it is not feasible to make trees that display the states of each of the relevant sites for all genes. However, I have included the phylogenetic trees in the relevant Additional files so that those can be opened in a program such as* FigTree *to examine the trees and map mutations to the branches.*

Minor issues –

Are all mutations with a signal reported in the violin graphs?

Author response: *Yes, in the violin plots, the points indicate all mutations with a signal of either differential or diversifying selection.*

Shouldn’t “beta-lactamase” be used throughout the paper rather than “lactamase”?

Author response: *Yes. In the revised version, I have made sure to fully write out “* *β* *-lactamase” rather than sometimes just saying “lactamase.”*

### Reviewer Report 3: Tal Pupko, Tel Aviv University, Israel

Reviewer summary –

Dr. Bloom is pushing forward an innovative idea: to integrate data from deep mutational scanning to improve the performance of the challenging task of identifying positively selected sites. To this end, he proposes a novel codon model that explicitly integrates such data within its parameters. I enjoyed the new concept, and I was convinced by the benefit of integrating such experimental data to improve dN/dS methods. I have some comments and suggestions to make the manuscript more accurate and informative.

Author response: *Thank you for the nice summary of the manuscript and the kind words.*

Reviewer recommendations to author –

All comments (major and minor) in the order they appear in the manuscript.

I felt that the first sentence is phrased in a non-scientific language. It is written that an important goal is to “identify genetic modifications that have led to interesting changes in phenotype.” Who decides what is interesting and what is not? I would rephrase to states that scientists want a better map between generic modifications and phenotypic variation.

Author response: *I have changed the word “interesting” to “evolutionarily significant,” which seems less subjective. However, I think some level of subjectivity is inherent in studying phenotypic changes. The researcher defines what is considered a phenotype that is worthy of study: for instance, in influenza virology we generally consider mutations that alter immunogenicity or host tropism to be “important,” and in the study of bacterial antibiotic resistance genes we typically consider as “important” mutations that enhance resistance to new drugs. But our choice to focus on those phenotypes is somewhat subjective. The approach in the current manuscript identifies sites that are evolving differently in nature than expected from experiments in the lab – but the choice to compare natural evolution to the “null model” of experiments in the lab is subjective, and is guided by the idea that pressures present in nature but absent in the lab are often relevant to phenotypes we consider “important” (for instance, immunogenicity for influenza, or extended-spectrum drug resistance for lactamase). I have elaborated on this point in the Discussion in the paragraph beginning “Does this reliance on experimental measurements make the approach less objective?”*

In page 2, it is written “for protein-coding genes, the most widely used methods for identifying specific sites of selection are built around the null model that non-synonymous and synonymous mutations should fix at equal rates.” I think this is inaccurate. Most biochemists interested to find purifying selective forces acting on their protein of interest do not use dN/dS methods. Instead, they use tools such as Consurf, which explicitly account for the physiochemical nature of the amino acids. Codon models are almost only used when explicitly searching for positive selection.

Author response: *This is a good point. I have changed “specific sites of selection” to “specific sites of positive selection.”*

Page 2, change “amino-acid mutation” to “non-synonymous mutation.”

Author response: *Thanks for catching this inconsistency in word usage, I have made this change.*

Page 2, it is claimed “detecting purifying selection as manifested by dN/dS < 1 points more to the naivety of the null model than unexpected biology”. As stated above, from a biochemical perspective it is highly important to know which sites are highly conserved and which ones are not. Such information is used, for example, for predicting which sites are buried and which are exposed to the solvent, which mutations are likely to cause diseases, and when the molecular mechanism of an enzyme is elucidated. Thus, when a codon model predicts and quantifies sites as being evolved under dN/dS < 1, this points to the fact that the model genuinely captures variation in purifying selective forces among amino acid sites. It does not point for a naivety of the model. Further, for dN/dS < 1, there is not a null model and an alternative model (which is not the case when searching for positive selection), so it is not clear what “null model” is in this statement.

Author response: *These are good points. I have simply removed the referenced sentence altogether, since it is unclear for the reason that reviewer notes. Specifically, the reviewer is correct that (depending on the question at hand), finding dN/dS <1 may be important (for instance, it is important for identifying disease-causing mutations, but not for finding viral immune escape mutations). However, it is true that many methods (such as FEL, FUBAR) for analyzing site-specific selection test both the alternative model dN/dS <1 and the alternative model dN/dS >1 against the null model dN/dS = 1, so for these methods there is a null model when testing dN/dS <1.*

Regarding the paragraph starting with “perhaps more importantly, dN/dS methods also have limited power to identify sites that have fixed adaptive mutations”: the term “fixed adaptive mutations” should be explained. Further, it is claimed that dN/dS methods have limited power, but only one example is provided (ref 10). As it is stated, the claim is not supported.

Author response: *I have changed the text from “also have limited power to identify” to “also can fail to identify.” This avoids a blanket statement that dN/dS methods lack power, since as the reviewer points out, I only cite a single example. However, I think that example justifies the statement that dN/dS methods can fail. And of course, the results in the current manuscript provide many more examples of sites of immune-escape or drug resistance mutations that are under positive selection but are not identified by a standard dN/dS method but are identified by the ExpCM.*

It is written that “the limitation of the null model that assumes equal rates of fixation of non-synonymous and synonymous mutations have become... ”. The standard codon models assume omega varies over sites according to a beta distribution (sometimes, a gamma distribution is assumed). By doing so, they assume that for most sites, the fixation rate of non-synonymous mutations is lower than the rate of synonymous mutations. Hence this statement in this sentence is inaccurate.

Author response: *I have re-written the text to read: “The limitations of simply comparing the rates of fixation of nonsynonymous and synonymous mutations have become especially glaring in light of deep mutational scanning experiments.” This statement along with the remainder of the paragraph effectively captures the key point that when sites are under very different levels of inherent constraint, a method that does not assign a different expectation of the expected constraint to each site will have difficulty identifying positive selection at constrained sites.*

In the last paragraph of the introduction it is written “But most sites strongly prefer one or a few amino acids; dN/dS methods do not offer a plausible null model for these sites”. This is again, inaccurate. There were many efforts to include amino acid preference with codon models. See for example (1) “An Empirical Codon Model for Protein Sequence Evolution”, a paper from the group of Nick Goldman; (2) “Empirical codon substitution matrix”, from the group of Gaston Gonnet; (3) “A Combined Empirical and Mechanistic Codon Model” from my own group; (4) A book chapter about empirical and semi empirical codon evolutionary models in the book “Codon Evolution: Mechanisms and Models” edited by David Liberles.

Author response: *I have re-worded the sentence in question. However, none of the references mentioned by the reviewer include* ***site-specific*** *constraints. They do treat different nonsynonymous substitutions differently, but this treatment is the same across sites (with the possible incorporation of a distributed rate parameter). Therefore, the stationary state of these models is homogeneous across sites (a rate parameter does not alter the model’s stationary state since it is simply a constant multiplying the transition matrix). The key difference of the ExpCM used here is that the treatment of each nonsynonymous substitution depends on the site, and so each site has a different stationary state. The re-wording of the sentence should better emphasize the key distinction.*

I had difficulties to understand figure 1A. To the best of my understanding, a comparison is made between amino acid preferences as measured by the deep mutations scanning of Stiffler et al. to the amino acid preferences in “nature”. However, it is not clear how the amino acid preferences in nature were computed. In addition, in Stiffler et al. several deep mutations scanning experiments were conducted. Which one is presented and why? It should also be better explained in which sites positive selection is expected, what is the “real” omega, what is the inferred omega of PAML.

Author response: *I have clarified the figure. I have added text to explain that the preferences shown in the figure are for the measurements from deep mutational scanning. None of the preferences are taken from natural sequence data – instead, there is just a comparison with which mutations are common in the naturally occurring sequences. I have added text to the legend explain that the Stiffler data is from the experiments with the highest concentration of ampicillin (this was previously explained only in the Methods). The violin plots show the P-value for* *ω* *>1 for each site computed using the FEL method; these P-values are shown rather than the* *ω* *value itself because site-specific estimates of* *ω* *are known to be numerically unreliable and so most methods focus on estimating the P-value (or posterior probability) of* *ω* *>1 rather than the numerical value of* *ω* *itself. The top column of text explains which sites are implicated in extended-spectrum antibiotic resistance; these are the ones that might reasonably be posited to be under positive selection.*

The methods are compared only to the “Goldman-Yang model” from 1994. In 1994 Goldman and Yang were the first codon model published, back to back with a paper by Muse and Gaut. I would suggest to use codon models that are used now, e.g., the M8-M8A model. Also, I am not sure that the real codon model proposed in Goldman and Yang (1994) was used. GY94, as stated in that paper in equation 3 includes explicit consideration of the amino acid type. Maybe the Muse and Gaut (1994) codon model was used instead? In light of these comments, I suggest that more details are provided for this figure so that readers can be convinced that a problem with standard codon models exists.

Author response: *This is a good point. The original Goldman-Yang paper [* 23 *] includes the possibility of weighting substitutions by amino-acid similarity (* *d*_*ij*_ *terms in their notation). In subsequent work [* 4 *], Yang and Goldman largely abandoned these weightings (i.e., made all* *d*_*ij*_ *) terms equal, and then defined various variants of these models (e.g., M0, M3, M8, etc). However, the literature commonly refers to all these model variants as “Goldman-Yang” style models, even though the reviewer is correct that they do not contain the weightings in the original Goldman-Yang paper. To clarify this, I have explicitly indicated that I have used specific M variants of the Goldman-Yang style models (e.g., M0) as defined in [* 4 *]. As far as the M8 model, I have chosen to instead use the M3 model for this paper. Like the M8 model, the M3 model allows multiple categories of* *ω* *. In earlier work using similar models [* 17 *], I have shown that the M3 and M8/M8a models give comparable performance.*

Figure 1, the P values are corrected for multiple testing using FDR. But in the legend it is written that Bonferroni correction is used. Maybe this should be better clarified?

Author response: *The tests were performed using an FDR. But in the case where there are no sites that are significant at an FDR of 0.05, the blue line indicates the P-value that would be needed by a single site to be significant with* *P* *=0.05 using a Bonferroni correction. This is equivalent to the FDR cutoff for just one site, since FDR and Bonferroni are identical when there is just one significant site. I have added text to clarify this.*

In the last paragraph of the introduction it is claimed that the goal is to detect sites under “differential selection for unexpected amino acids”. Is this identical with the goal of “detecting sites evolving under positive selection”? There are many other works that aim to detect selection shifts (e.g., the extensive literature on covarion models). This is not the same as to detect positive selection.

Author response: *This is a valid point, although as the results in the manuscript show, in many cases the sites of differential selection turn out to be sites of adaptive mutations. I have added a sentence in the last paragraph of the Introduction emphasizing that this strategy “seeks to identify sites that are evolving differently in nature than expected from constraints measured in the lab.” As I think the subsequent results show, in many cases these sites turn out to be ones that have fixed immune-escape or drug-resistance mutations that would typically be envisioned as having arisen from positive selection for adaptation.*

The first part of the results is dedicated to a description of the ExpCM model. It is written: “The ExpCM used here are similar but not identical to those in [16,17]”. However, the differences are not explicitly stated nor are the reasons for changing the model. I suggest making this statement more explicit.

Author response: *Good suggestion. I have clarified in the text how the ExpCM differ. They differ by including the* *ω* *term, and by using a slightly different model (an HKY85 model) for handling the nucleotide mutation rates.*

In Equation 5, variability in the synonymous rate among sites is included. Why not to include it already in the null model, i.e., Equation 3 (see also “Towards realistic codon models: among site variability and dependency of synonymous and non-synonymous rates.” [77])? Also, when comparing to the standard model, how can one know the contribution of adding the data from the deep mutation scanning versus the contribution to power stemming from adding a component of synonymous variation over sites? At any rate, a more elaborate way to test for deviation from the null model, would be to generate an alternative model for all sites that would allow omega to vary across sites. Then to estimate, for example, the posterior expectation of omega for each site. Such an approach would allow for example to account for uncertainty in model parameters, by adding a BEB (Bayes Empirical Bayesian) component.

Author response: *The synonymous rate variation is not included in the gene-wide model, but is included in the site-specific fitting to test for diversifying selection. Specifically, when fitting Equation 5, the null model is to fit just* *μ*_*r*_ *(synonymous rate) and fix* *ω*_*r*_ *=1, while the alternative model is to fit both* *ω*_*r*_ *and* *μ*_*r*_ *, so this ensures that any improvement in site-specific estimation is not due to the synonymous rate. The reason I have taken this approach is that it is used in the FEL approaches (and many other standard approaches) to which the comparisons are made (see [* 27 *]). Therefore, all the comparisons between the null and alternative models of both the ExpCM and more standard GY94-style models handle synonymous rate variation comparably, ensuring an apples-to-apples comparison*.

*The reviewer is correct that real biological processes might involve synonymous rate variation as well. This possibility is nicely discussed in the reviewer’s own paper on the topic [* 77 *]. Therefore, in the concluding paragraph of the Discussion, I have cited [* 77 *] and added mention of how better incorporating synonymous rate variation might be one possible way to extend/improve ExpCM. Note however that the pros and cons of incorporating synonymous rate variation remain a topic of active debate [* 92 *], although I tend to side with the reviewer [* 77 *] and others [* 27 *] that incorporating such variation is beneficial.*

*I agree that using an empirical Bayes approach is an alternative framework to try, although again the relative pros and cons of these so-called “random effects” methods versus their “fixed effects” alternatives remains a topic of active debate [* 5,53,93 *]. I discuss this issue and the possibility of extending ExpCM to an empirical Bayes framework in the Results paragraph beginning “A variety of other* *d* *N*/*d* *S* *methods have also been developed.”*

Below equation 5 it is written that the key statistic is not omega itself, but rather the P-value. I don’t think the P value is the statistic, but rather, the log likelihood ratio.

Author response: *I have clarified this point by stating that the key statistic is the difference in log likelihoods (the likelihood ratio), from which a P-value can be computed.*

The balancing term introduced in equation 7 seems to be equivalent to assuming a specific prior distribution over the amino acid distributions. However, the connection with a prior distribution is very implicit. I suggest moving to a Bayesian approach and thus making this prior assumption explicit. If this is not feasible in the current version of the manuscript, please consider stating this link to an implicit prior.

Author response: *In a Bayesian approach, this equation would be the equivalent of a prior. However, since the current manuscript uses a maximum-likelihood approach, the equation is better thought of as a regularization term, since we are not actually sampling from the posterior established by the likelihood and prior, but rather simply maximizing the likelihood subject to the regularization established by Equation 7. I have added a sentence making this link between regularization and a Bayesian prior. Given the current computational implementation, it is not straightforward to move the analysis to a Bayesian approach. But as mentioned in the response two before this one, this is an interesting area for future work, and one that I discuss in the manuscript.*

When comparing the power of the ExpCM method to “GY94”, it seems to me that there is also a difference in the false positive rate.

Author response: *This is true. The ExpCM has a false-discovery rate that is close to what is expected given the FDR of 0.05, while the GY94 has a lower false-discovery rate but also a much higher false negative rate.*

Minor comments -

Consider reducing the number of additional files and move some info into the main text.

Author response: *I admit there are a lot of additional files. However, for both an earlier version of this manuscript submitted elsewhere and the first version I posted on bioRxiv, I received exactly the opposite complaint that there were too many figures that would be better moved to additional files! So I think I am going to keep it as is, knowing that in the final published version (which will have working links) it will be much easier for the reader to access the additional files.*

## Additional files

Additional file 1 Graph of the function used to regularize the *Δ* *π*_*r*,*a*_ values when inferring differential selection. The log of the regularization defined by Eq. 7 is a sum of terms like this taken over all differential preferences at a site. This regularization has the property that the marginal cost of shifting *Δ* *π*_*r*,*a*_ away from zero is initially steep but then flattens somewhat as *Δ* *π*_*r*,*a*_ becomes large. This corresponds to the intuition that most sites will be evolving as expected (and so have *Δ* *π*_*r*,*a*_∼0), but a few sites might be under strong differential selection. This plot uses *C*_1_=150 and *C*_2_=0.5. (PDF 284 kb)

Additional file 2 Site-specific amino-acid preferences for Gal4. Shown are the preferences experimentally measured by [31] for the DNA-binding domain of yeast Gal4, re-scaled by the stringency parameter *β*= 0.82 from Table 2. (PDF 25 kb)

Additional file 3 Site-specific amino-acid preferences for NP. Site-specific amino-acid preferences for influenza NP. Shown are the preferences experimentally reported in [32] for the average of the measurements on the A/PR/8/1934 and A/Aichi/2/1968 strains, re-scaled by the stringency parameter *β*=2.43 from Table 2. (PDF 68 kb)

Additional file 4 Site-specific amino-acid preferences for HA. Shown are the preferences experimentally measured by [33] for influenza HA (A/WSN/1933, H1N1 strain), re-scaled by the stringency parameter *β*= 1.61 from Table 2. The residues are numbered according to the H3 numbering scheme (the one used in PDB 4HMG), and data are only shown for sites in the HA ectodomain (residues present in the crystal structure in PDB 4HMG). (PDF 100 kb)

Additional file 5 Simulations validate the statistical approach used to identify diversifying selection. Using the actual ExpCM parameters for NP in Table 2 except fixing *ω*=1 for all sites except for those selected to be simulated under diversifying selection, I used pyvolve [46] to simulate 40 alignments along the tree inferred from the actual NP sequences. For each simulation, I randomly selected 5 sites to place under diversifying selection, with *ω*_*r*_ values ranging from 1 (no diversifying selection) to 30 (very strong diversifying selection). I then analyzed the data using phydms in the same way that the actual data were analyzed. Sites were called as being under significant diversifying selection using the false discovery rates (FDRs) indicated in the figure. The top panel shows that ExpCM greatly outperformed the FEL-like GY94 method at identifying true positives. The bottom panel shows that the Benjamini-Hocbherg [28] procedure effectively controls the fraction of false discoveries among the sites called as being under diversifying selection using ExpCM. The Benjamini-Hochberg procedure may be slightly too conservative for ExpCM (for every value of *ω*_*r*_ the actual rate of false discoveries is slightly below the FDR), but the differences seem modest. The computer code to perform these simulations is in Additional file 17. (PDF 150 kb)

Additional file 6 This figure is same as Fig. 3 a but also includes an analysis with MEME [6] as implemented in HyPhy [7]. MEME reports the *P*-value that a site has *d* *N*/*d* *S*>1 on at least some branches of the tree. As can be seen from this figure, MEME is somewhat more powerful than the GY94-based FEL approach, presumably because some sites are only under episodic diversifying selection. While the GY94-based FEL approach identifies no sites of diversifying selection, MEME identifies one site of diversifying selection in *β*-lactamase and one site in NP. However, MEME still identifies fewer sites for all genes than the ExpCM. (PDF 287 kb)

Additional file 7 This figure shows the distribution over sites of the Kullback-Leibler divergence of the experimentally measured amino-acid preferences from the alignment frequencies. Note that the Kullback-Leibler divergence does *not* take phylogeny into account, and so will be confounded the incomplete sampling of potentially tolerated amino acids by natural evolution. The distribution of per-site Kullback-Leibler divergences shown here lacks the biologically sensible features of the differential selection computed in a phylogenetic framework and shown in Fig. 3 b. For instance, Gal4 has many sites with very high Kullback-Leibler divergence even though on biological grounds we expect it to be evolving mostly in the absence of positive selection. In contrast, *β*-lactamase and NP tend to have lower Kullback-Leibler divergence even though we know that they evolve under selection for adaptive mutations that confer drug resistance or immune escape. The biologically unreasonable distribution of Kullback-Leibler divergences shown in this plot are probably due to the failure of the Kullback-Leibler divergence to account for phylogeny, which may in turn make the results highly sensitive to uneven phylogenetic sampling and differences in the total sequence divergence spanned by the alignments (see Table 1). The Kullback-Leibler divergence was computed using logarithms taken to the base two. (PDF 86 kb)

Additional file 8 Site-specific selection on Gal4 inferred with the experimentally informed models. This figure is equivalent to Fig. 4 but for Gal4. (PDF 252 kb)

Additional file 9 Site-specific selection on NP inferred with the experimentally informed models. This figure is equivalent to Fig. 4 but for NP. (PDF 278 kb)

Additional file 10 Site-specific selection on HA inferred with the experimentally informed models. This figure is equivalent to Fig. 4 but for HA. (PDF 282 kb)

Additional file 11 The data and code for running the analysis for Gal4. This is a 7-Zip file containing an iPython notebook and the relevant data files. (7z 1260 kb)

Additional file 12 The data and code for running the analysis for lactamase. This is a 7-Zip file containing an iPython notebook and the relevant data files. (7z 2692 kb)

Additional file 13 The data and code for running the analysis for NP. This is a 7-Zip file containing an iPython notebook and the relevant data files. (7Z 2910 kb)

Additional file 14 The data and code for running the analysis for HA. This is a 7-Zip file containing an iPython notebook and the relevant data files. (7Z 3000 kb)

Additional file 15 The data and code for running the pyvolve simulations. This is a 7-Zip file containing an iPython notebook and the relevant data files. (7Z 7450 kb)

Additional file 16 Clarification of subtleties in the relationship between amino-acid preferences and substitution model equilibrium frequencies. Figure 1 shows the experimentally measured amino-acid preferences and the equilibrium frequencies of the GY94 model. The equilibrium frequencies of the experimentally informed codon models (ExpCM) are given by Eq. 4, and are similar but not identical to the preferences: the ExpCM equilibrium frequencies are also influenced by the unequal number of codons per amino acid, nucleotide mutation biases, and the stringency parameter *β*. The equilibrium frequencies of the GY94 model already account for the codon/mutation factors. To clarify these distinctions, this figure shows the preferences and equilibrium frequencies of the ExpCM model, and the “all-equal” amino-acid preferences that would lead to the equilibrium frequencies of the GY94 model if the nucleotide frequency parameters in that model are construed as representing mutation-level rather than selection-level processes. Note that the logo plots show the amino-acid frequencies implied by the equilibrium codon frequencies (i.e. the sum of the frequencies of all encoding codons for each amino acid). (PDF 76 kb)

Additional file 17 The results of the *d* *N*/*d* *S* analysis are qualitatively similar when using HyPhy rather than phydms. This figure shows the same data as that in Fig. 3 a, but also includes the results of a *d* *N*/*d* *S* analysis using the fixed effects likelihood (FEL) method implemented in HyPhy [7]. The results are not identical to the phydms GY94 results because the HyPhy implementation differs slightly from the phydms implementation: HyPhy performs the *d* *N*/*d* *S* analysis using the substitution model of [102] rather than GY94, and infers a neighbor-joining tree with a nucleotide substitution model rather than a maximum-likelihood tree using a codon model. Nonetheless, the results of the HyPhy FEL analysis are highly similar to those of the phydms GY94 analysis, both in terms of the overall distribution of results and in terms of the values for the specific indicated sites. The point markers represent the same sites as in Fig. 3. (PDF 236 kb)

## Abbreviations

AIC: Akaike Information Criterion
ExpCM: Experimentally informed codon model
GY94: Goldman Yang 1994 substitution model
HA: Hemagglutinin
NP: Nucleoprotein
