# Supplementary figures and images for: Identification of positive selection in genes is greatly improved by using experimentally informed site-specific models
Source: Biol Direct. 2017 Jan 17;12:1. doi: 10.1186/s13062-016-0172-z (PMC5240389; doi:10.1186/s13062-016-0172-z)

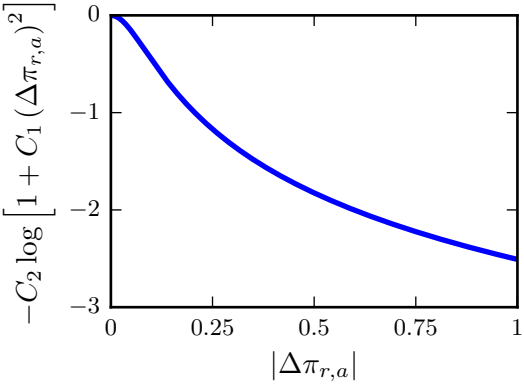

Supplement: Additional file 1 — Graph of the function used to regularize the Δ π r,a values when inferring differential selection. The log of the regularization defined by Eq. 7 is a sum of terms like this taken over all differential preferences at a site. This regularization has the property that the marginal cost of shifting Δ π r,a away from zero is initially steep but then flattens somewhat as Δ π r,a becomes large. This corresponds to the intuition that most sites will be evolving as expected (and so have Δ π r,a∼0), but a few sites might be under strong differential selection. This plot uses C 1=150 and C 2=0.5. (PDF 284 kb) [file 13062_2016_172_MOESM1_ESM.pdf]

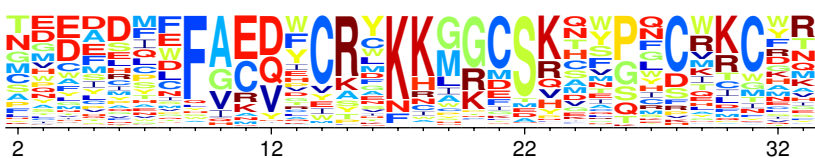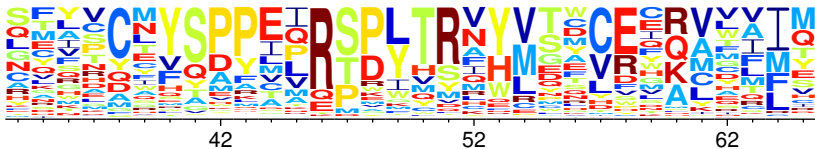

Supplement: Additional file 2 — Site-specific amino-acid preferences for Gal4. Shown are the preferences experimentally measured by [31] for the DNA-binding domain of yeast Gal4, re-scaled by the stringency parameter β= 0.82 from Table 2. (PDF 25 kb) [file 13062_2016_172_MOESM2_ESM.pdf]

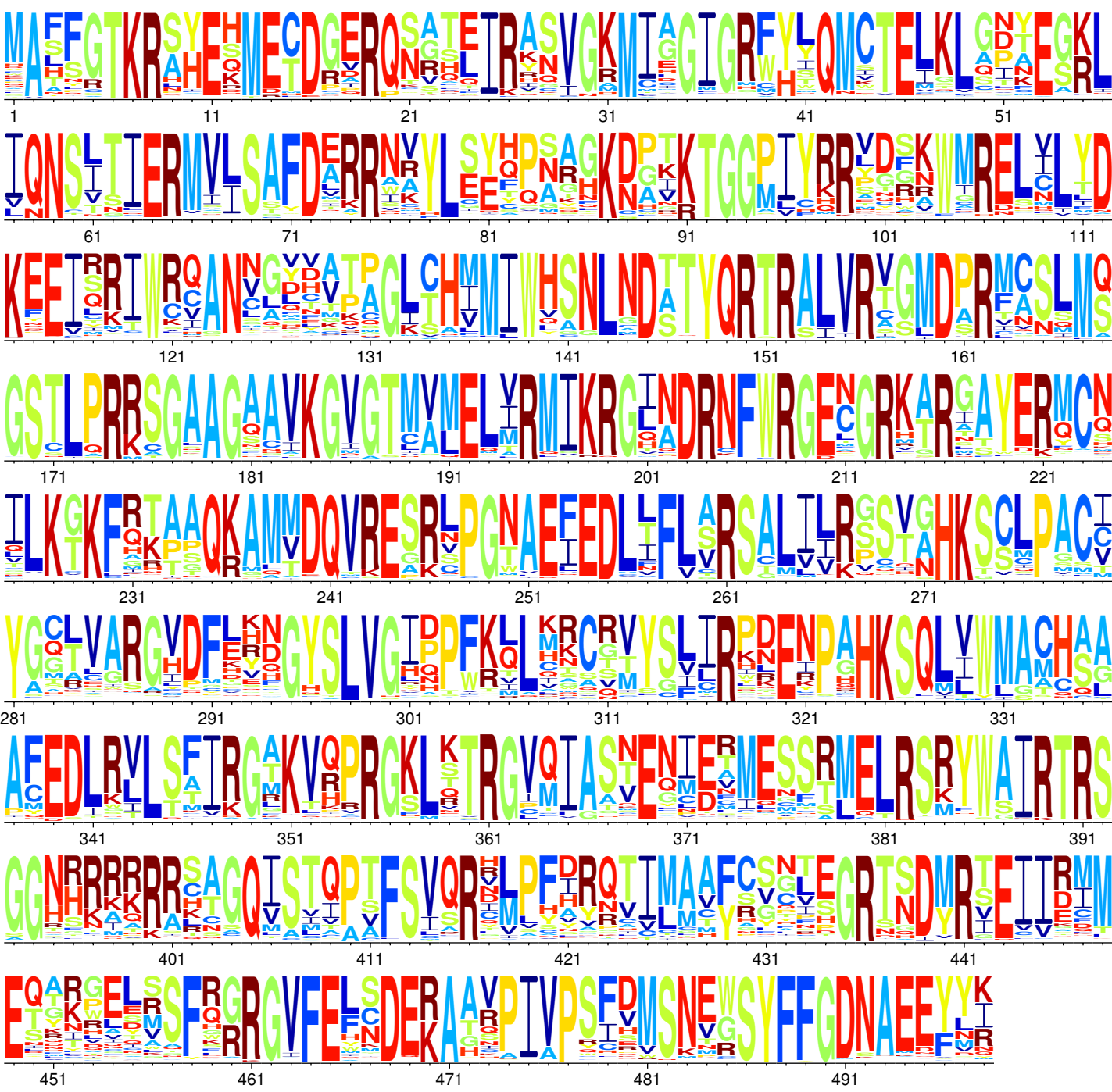

Supplement: Additional file 3 — Site-specific amino-acid preferences for NP. Site-specific amino-acid preferences for influenza NP. Shown are the preferences experimentally reported in [32] for the average of the measurements on the A/PR/8/1934 and A/Aichi/2/1968 strains, re-scaled by the stringency parameter β=2.43 from Table 2. (PDF 68 kb) [file 13062_2016_172_MOESM3_ESM.pdf]

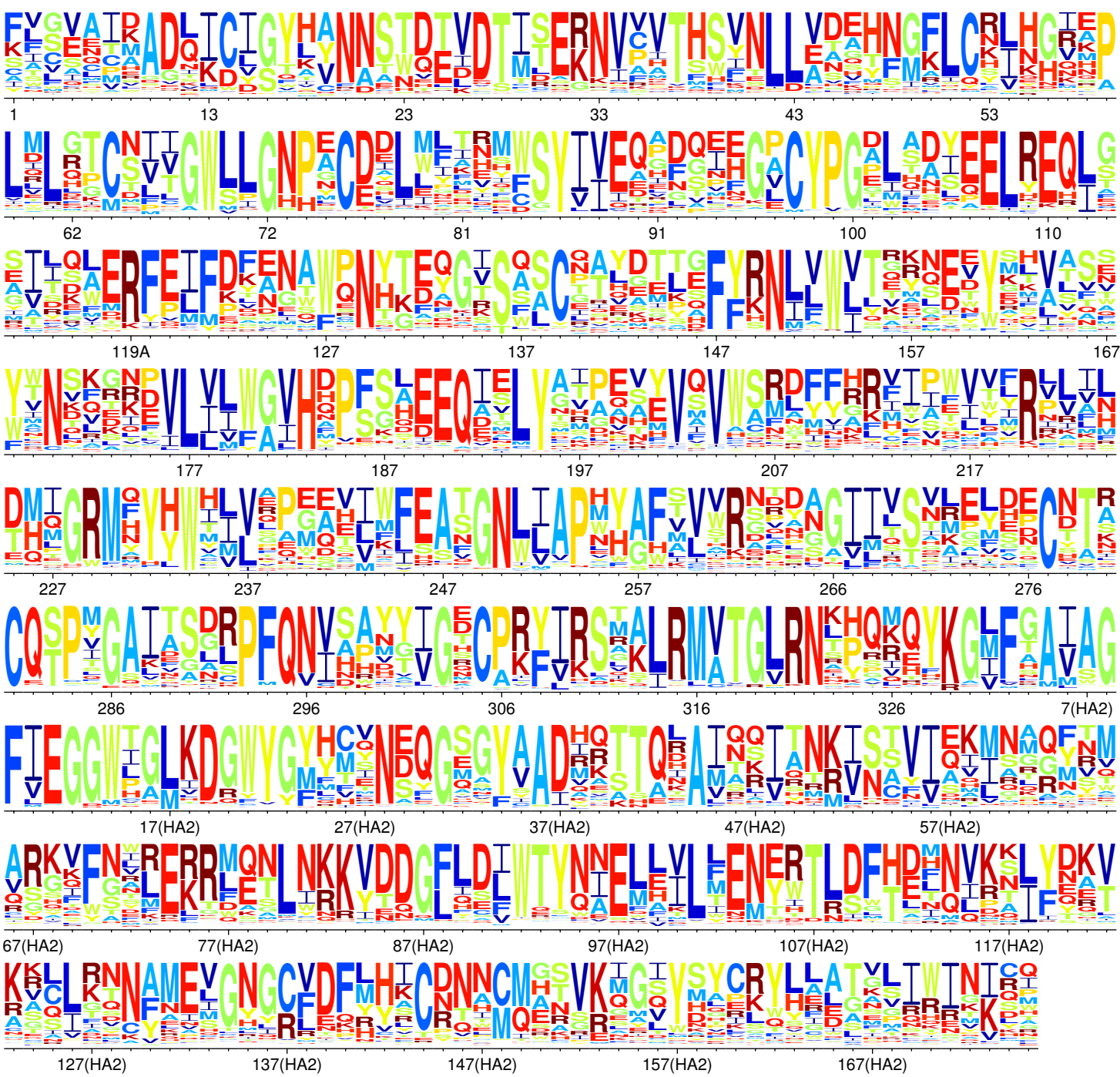

Supplement: Additional file 4 — Site-specific amino-acid preferences for HA. Shown are the preferences experimentally measured by [33] for influenza HA (A/WSN/1933, H1N1 strain), re-scaled by the stringency parameter β= 1.61 from Table 2. The residues are numbered according to the H3 numbering scheme (the one used in PDB 4HMG), and data are only shown for sites in the HA ectodomain (residues present in the crystal structure in PDB 4HMG). (PDF 100 kb) [file 13062_2016_172_MOESM4_ESM.pdf]

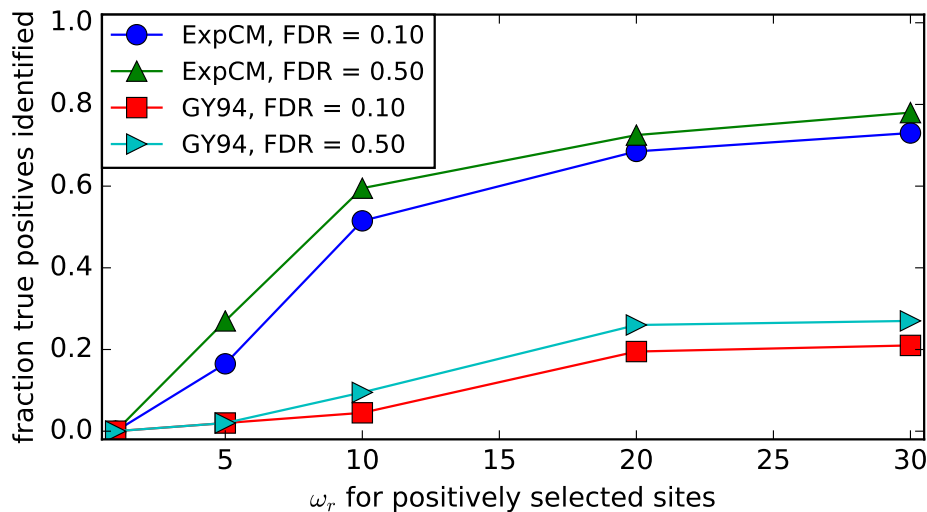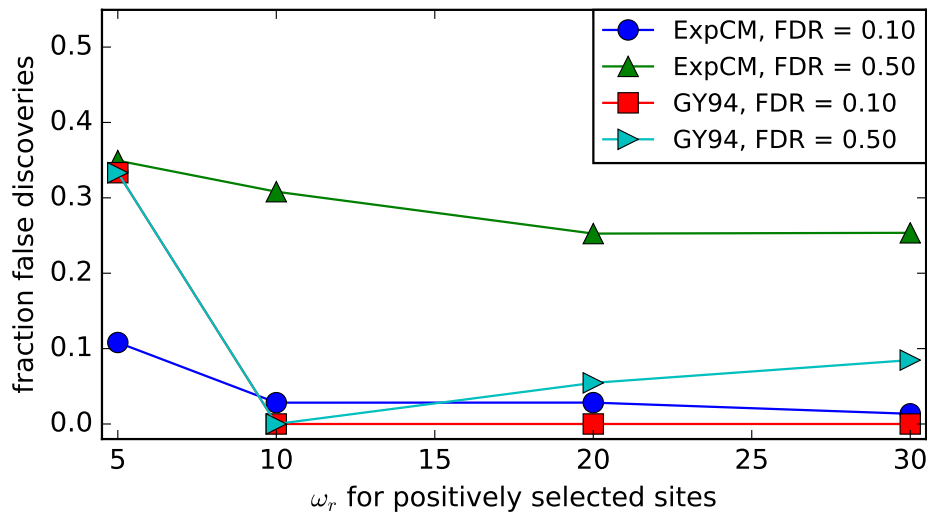

Supplement: Additional file 5 — Simulations validate the statistical approach used to identify diversifying selection. Using the actual ExpCM parameters for NP in Table 2 except fixing ω=1 for all sites except for those selected to be simulated under diversifying selection, I used pyvolve [46] to simulate 40 alignments along the tree inferred from the actual NP sequences. For each simulation, I randomly selected 5 sites to place under diversifying selection, with ω r values ranging from 1 (no diversifying selection) to 30 (very strong diversifying selection). I then analyzed the data using phydms in the same way that the actual data were analyzed. Sites were called as being under significant diversifying selection using the false discovery rates (FDRs) indicated in the figure. The top panel shows that ExpCM greatly outperformed the FEL-like GY94 method at identifying true positives. The bottom panel shows that the Benjamini-Hocbherg [28] procedure effectively controls the fraction of false discoveries among the sites called as being under diversifying selection using ExpCM. The Benjamini-Hochberg procedure may be slightly too conservative for ExpCM (for every value of ω r the actual rate of false discoveries is slightly below the FDR), but the differences seem modest. The computer code to perform these simulations is in Additional file 17. (PDF 150 kb) [file 13062_2016_172_MOESM5_ESM.pdf]

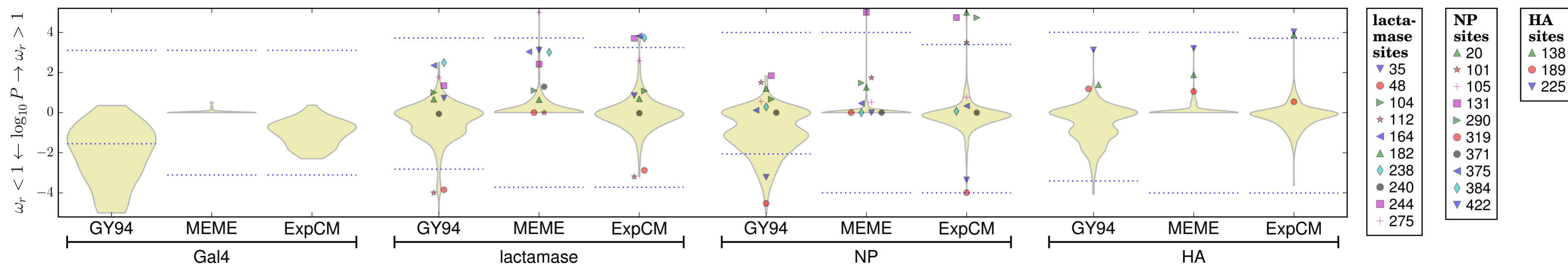

Supplement: Additional file 6 — This figure is same as Fig. 3 a but also includes an analysis with MEME [6] as implemented in HyPhy [7]. MEME reports the P-value that a site has d N/d S>1 on at least some branches of the tree. As can be seen from this figure, MEME is somewhat more powerful than the GY94-based FEL approach, presumably because some sites are only under episodic diversifying selection. While the GY94-based FEL approach identifies no sites of diversifying selection, MEME identifies one site of diversifying selection in β-lactamase and one site in NP. However, MEME still identifies fewer sites for all genes than the ExpCM. (PDF 287 kb) [file 13062_2016_172_MOESM6_ESM.pdf]

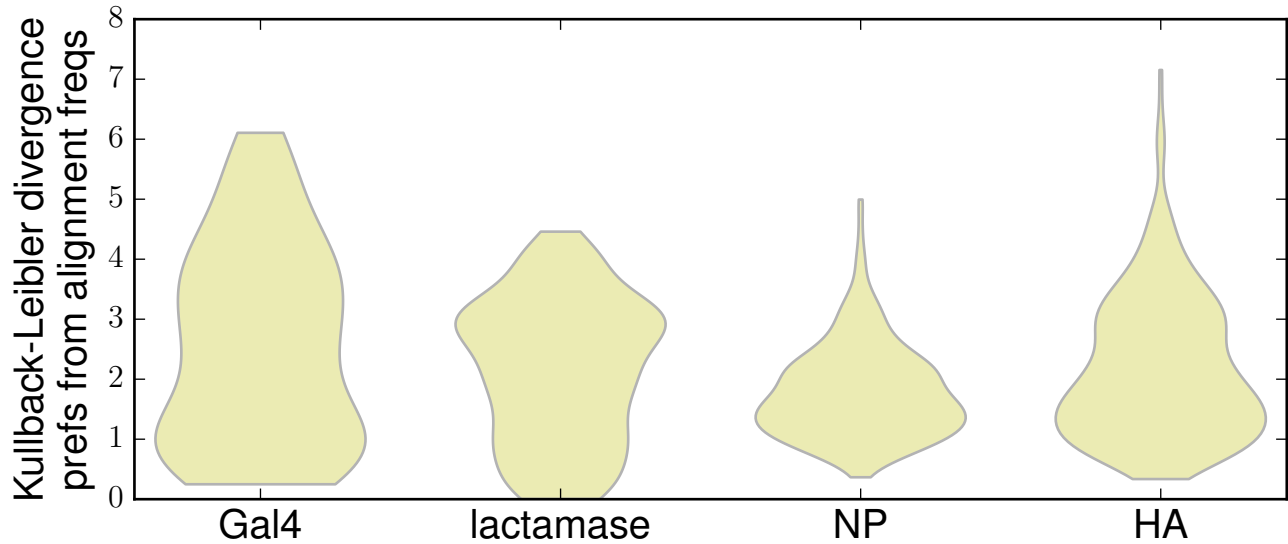

Supplement: Additional file 7 — This figure shows the distribution over sites of the Kullback-Leibler divergence of the experimentally measured amino-acid preferences from the alignment frequencies. Note that the Kullback-Leibler divergence does not take phylogeny into account, and so will be confounded the incomplete sampling of potentially tolerated amino acids by natural evolution. The distribution of per-site Kullback-Leibler divergences shown here lacks the biologically sensible features of the differential selection computed in a phylogenetic framework and shown in Fig. 3 b. For instance, Gal4 has many sites with very high Kullback-Leibler divergence even though on biological grounds we expect it to be evolving mostly in the absence of positive selection. In contrast, β-lactamase and NP tend to have lower Kullback-Leibler divergence even though we know that they evolve under selection for adaptive mutations that confer drug resistance or immune escape. The biologically unreasonable distribution of Kullback-Leibler divergences shown in this plot are probably due to the failure of the Kullback-Leibler divergence to account for phylogeny, which may in turn make the results highly sensitive to uneven phylogenetic sampling and differences in the total sequence divergence spanned by the alignments (see Table 1). The Kullback-Leibler divergence was computed using logarithms taken to the base two. (PDF 86 kb) [file 13062_2016_172_MOESM7_ESM.pdf]

$\omega_r < 1 \leftarrow \log_{10} P \text{ for } \omega \rightarrow \omega_r > 1$

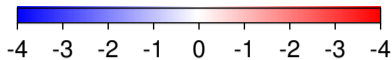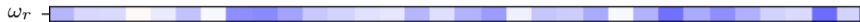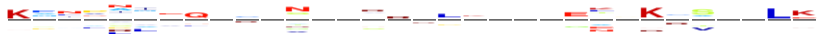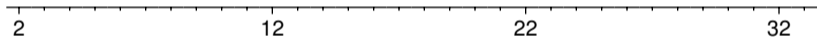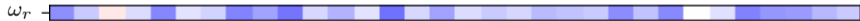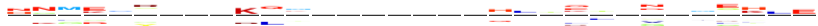

42

52

62

Supplement: Additional file 8 — Site-specific selection on Gal4 inferred with the experimentally informed models. This figure is equivalent to Fig. 4 but for Gal4. (PDF 252 kb) [file 13062_2016_172_MOESM8_ESM.pdf]

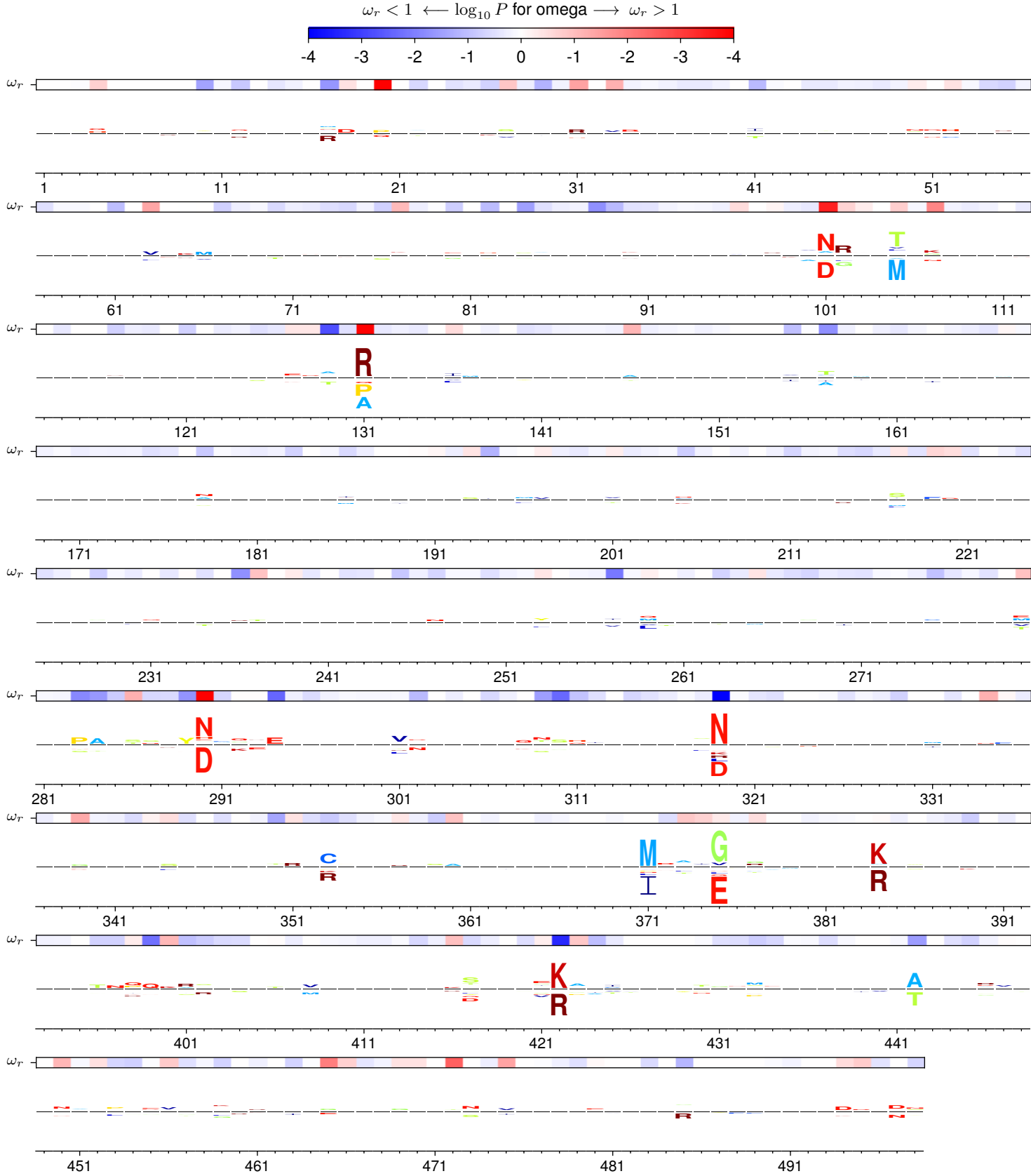

Supplement: Additional file 9 — Site-specific selection on NP inferred with the experimentally informed models. This figure is equivalent to Fig. 4 but for NP. (PDF 278 kb) [file 13062_2016_172_MOESM9_ESM.pdf]

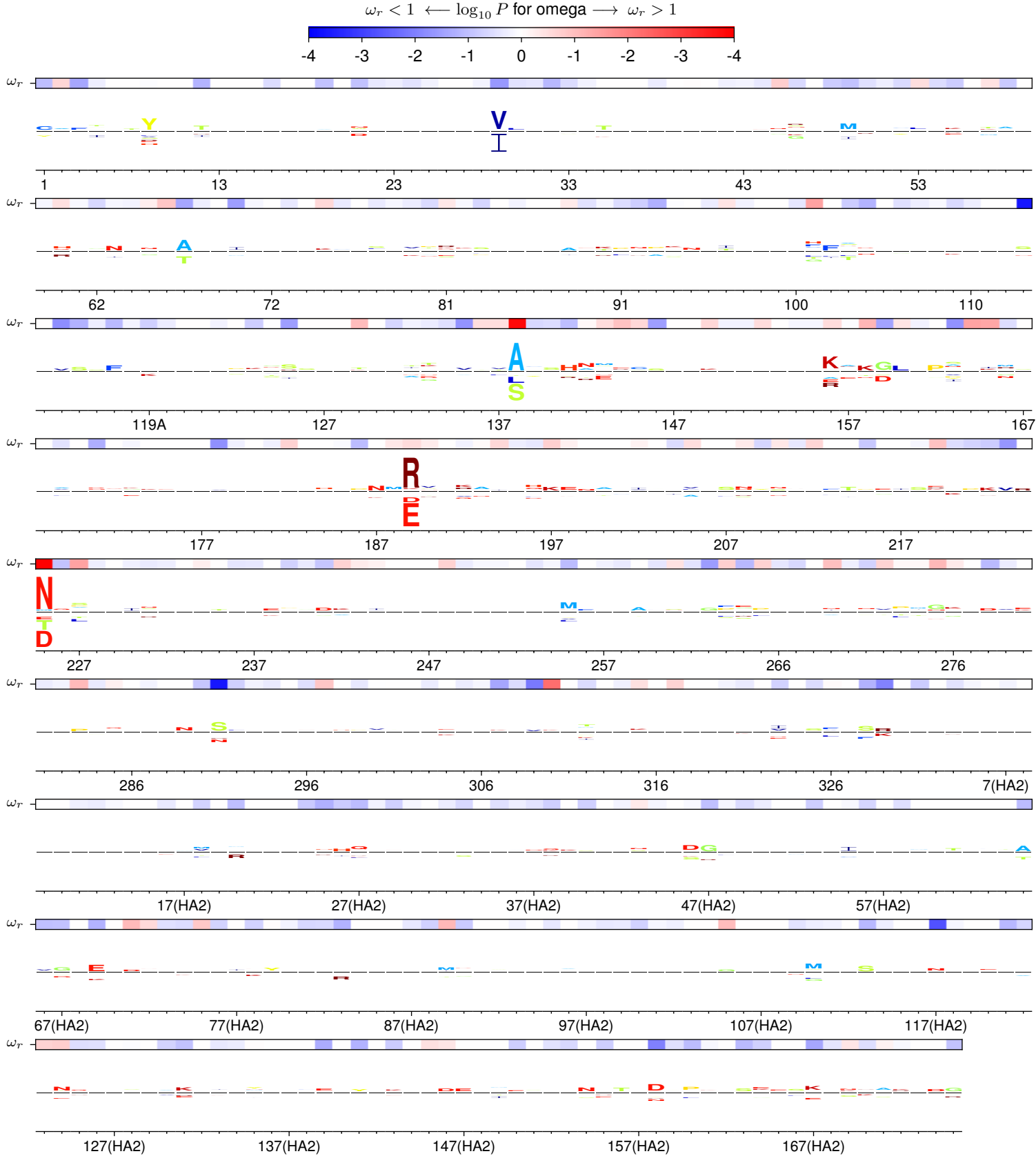

Supplement: Additional file 10 — Site-specific selection on HA inferred with the experimentally informed models. This figure is equivalent to Fig. 4 but for HA. (PDF 282 kb) [file 13062_2016_172_MOESM10_ESM.pdf]

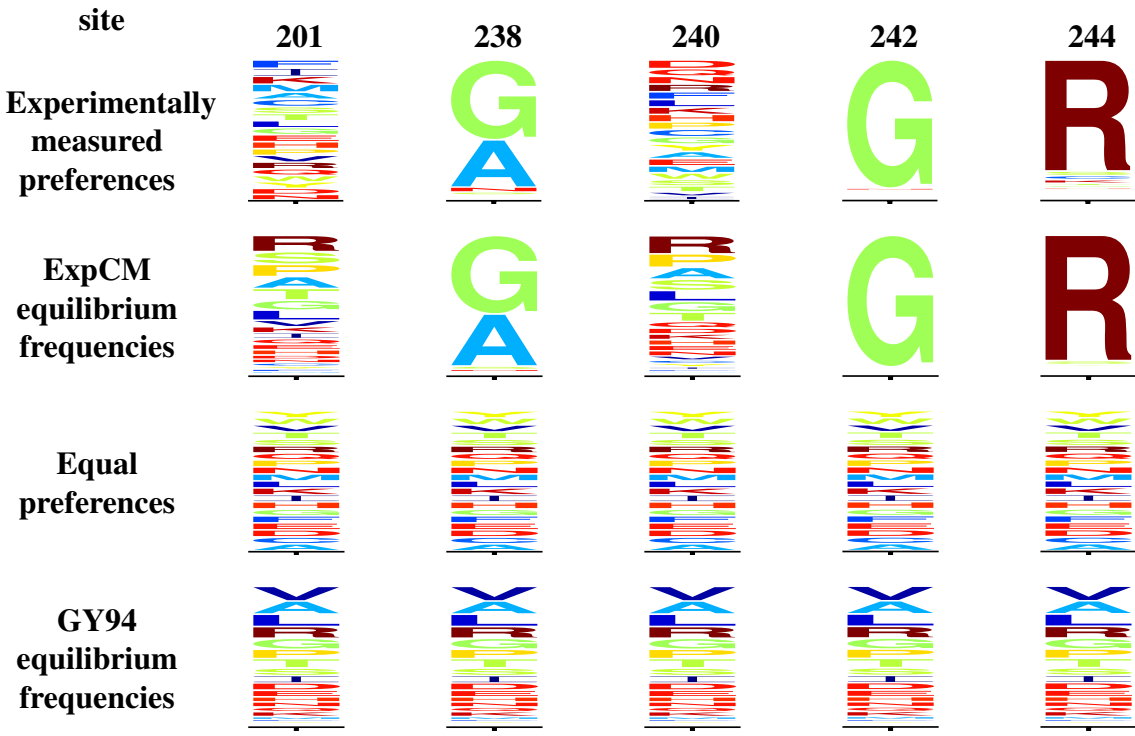

Supplement: Additional file 16 — Clarification of subtleties in the relationship between amino-acid preferences and substitution model equilibrium frequencies. Figure 1 shows the experimentally measured amino-acid preferences and the equilibrium frequencies of the GY94 model. The equilibrium frequencies of the experimentally informed codon models (ExpCM) are given by Eq. 4, and are similar but not identical to the preferences: the ExpCM equilibrium frequencies are also influenced by the unequal number of codons per amino acid, nucleotide mutation biases, and the stringency parameter β. The equilibrium frequencies of the GY94 model already account for the codon/mutation factors. To clarify these distinctions, this figure shows the preferences and equilibrium frequencies of the ExpCM model, and the “all-equal” amino-acid preferences that would lead to the equilibrium frequencies of the GY94 model if the nucleotide frequency parameters in that model are construed as representing mutation-level rather than selection-level processes. Note that the logo plots show the amino-acid frequencies implied by the equilibrium codon frequencies (i.e. the sum of the frequencies of all encoding codons for each amino acid). (PDF 76 kb) [file 13062_2016_172_MOESM16_ESM.pdf]

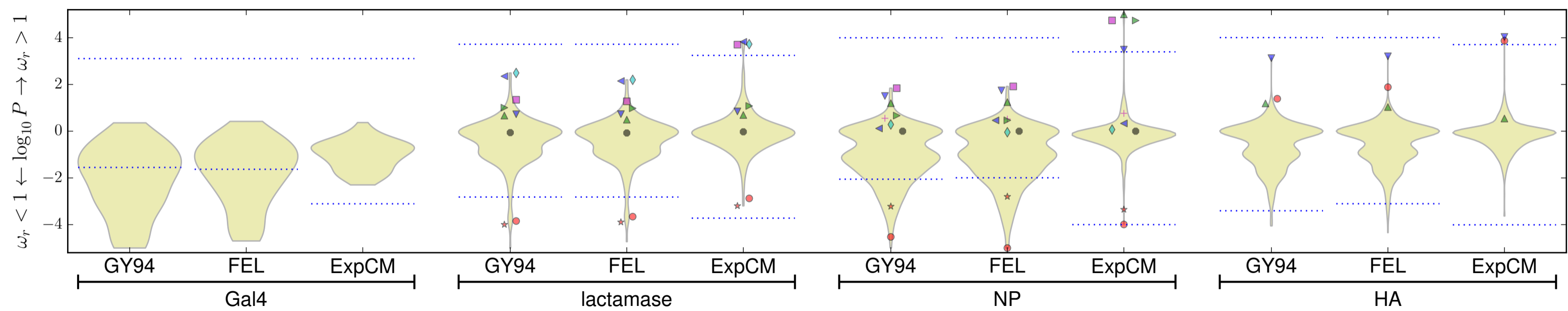

Supplement: Additional file 17 — The results of the d N/d S analysis are qualitatively similar when using HyPhy rather than phydms. This figure shows the same data as that in Fig. 3 a, but also includes the results of a d N/d S analysis using the fixed effects likelihood (FEL) method implemented in HyPhy [7]. The results are not identical to the phydms GY94 results because the HyPhy implementation differs slightly from the phydms implementation: HyPhy performs the d N/d S analysis using the substitution model of [102] rather than GY94, and infers a neighbor-joining tree with a nucleotide substitution model rather than a maximum-likelihood tree using a codon model. Nonetheless, the results of the HyPhy FEL analysis are highly similar to those of the phydms GY94 analysis, both in terms of the overall distribution of results and in terms of the values for the specific indicated sites. The point markers represent the same sites as in Fig. 3. (PDF 236 kb) [file 13062_2016_172_MOESM17_ESM.pdf]
